# Challenges involved in cell therapy for Parkinson’s disease using human pluripotent stem cells

**DOI:** 10.3389/fcell.2023.1288168

**Published:** 2023-10-11

**Authors:** Heechang Moon, Bokwang Kim, Inbeom Kwon, Yohan Oh

**Affiliations:** ^1^ Department of Biomedical Science, Graduate School of Biomedical Science and Engineering, Hanyang University, Seoul, Republic of Korea; ^2^ Department of Medicine, College of Medicine, Hanyang University, Seoul, Republic of Korea; ^3^ Department of Biochemistry and Molecular Biology, College of Medicine, Hanyang University, Seoul, Republic of Korea; ^4^ Hanyang Institute of Bioscience and Biotechnology, Hanyang University, Seoul, Republic of Korea; ^5^ Hanyang Institute of Advanced BioConvergence, Hanyang University, Seoul, Republic of Korea

**Keywords:** cell therapy, human pluripotent stem cells, midbrain dopaminergic progenitors, neurodegenerative diseases, Parkinson’s disease

## Abstract

Neurons derived from human pluripotent stem cells (hPSCs) provide a valuable tool for studying human neural development and neurodegenerative diseases. The investigation of hPSC-based cell therapy, involving the differentiation of hPSCs into target cells and their transplantation into affected regions, is of particular interest. One neurodegenerative disease that is being extensively studied for hPSC-based cell therapy is Parkinson’s disease (PD), the second most common among humans. Various research groups are focused on differentiating hPSCs into ventral midbrain dopaminergic (vmDA) progenitors, which have the potential to further differentiate into neurons closely resembling DA neurons found in the substantia nigra pars compacta (SNpc) after transplantation, providing a promising treatment option for PD. In *vivo* experiments, where hPSC-derived vmDA progenitor cells were transplanted into the striatum or SNpc of animal PD models, the transplanted cells demonstrated stable engraftment and resulted in behavioral recovery in the transplanted animals. Several differentiation protocols have been developed for this specific cell therapy. However, the lack of a reliable live-cell lineage identification method presents a significant obstacle in confirming the precise lineage of the differentiated cells intended for transplantation, as well as identifying potential contamination by non-vmDA progenitors. This deficiency increases the risk of adverse effects such as dyskinesias and tumorigenicity, highlighting the importance of addressing this issue before proceeding with transplantation. Ensuring the differentiation of hPSCs into the target cell lineage is a crucial step to guarantee precise therapeutic effects in cell therapy. To underscore the significance of lineage identification, this review focuses on the differentiation protocols of hPSC-derived vmDA progenitors developed by various research groups for PD treatment. Moreover, *in vivo* experimental results following transplantation were carefully analyzed. The encouraging outcomes from these experiments demonstrate the potential efficacy and safety of hPSC-derived vmDA progenitors for PD cell therapy. Additionally, the results of clinical trials involving the use of hPSC-derived vmDA progenitors for PD treatment were briefly reviewed, shedding light on the progress and challenges faced in translating this promising therapy into clinical practice.

## 1 Introduction

Parkinson’s disease (PD) is the second most common neurodegenerative disease in humans. It is caused by the specific loss of dopaminergic (DA) neurons in the substantia nigra pars compacta (SNpc) of the midbrain. PD is characterized by motor symptoms, such as tremor, muscular stiffness, and bradykinesia, but it is also associated with cognitive impairment, sleep disturbances, depression, and a weakened sense of smell (Poewe et al., 2017). Various methods have been used to treat PD. Drug therapy, deep brain stimulation (DBS), gene therapy, and cell therapy are currently available treatments. Levodopa, dopamine agonists, and monoamine oxidase-B (MAO-B) inhibitors are representative drugs used in an attempt to increase low dopamine levels in PD patients (Connolly and Lang, 2014). These drug therapies are known to improve the motor symptoms of patients, but long-term treatment with levodopa or dopamine agonists can worsen the patient’s symptoms due to drug tolerance and neurotoxicity. Additionally, nausea, daytime somnolence, and edema are possible adverse effects of these treatments (Connolly and Lang, 2014). DBS is a surgical therapy that alleviates symptoms by inserting electrodes into movement-controlling regions of the brain, such as the subthalamic nucleus (STN) or globus pallidus internus (GPi), for electrical stimulation (Bronstein et al., 2011). Patients who have acquired medication resistance due to long-term pharmacological therapy may benefit from DBS. The advantage of DBS is that the patient may reduce their medicine dosage and switch the electrodes on and off as required. As the DBS-implanted brain areas are also involved with emotions, adverse effects, such as emotional disorders and manic responses, may result in psychiatric issues.

Currently, the above-mentioned therapies may improve PD symptoms, but a fundamental cure is not yet available. Consequently, diverse treatment methods are being researched. One of these, gene therapy, aims to fix genetic mutations in familial PD, and numerous therapeutic techniques have been proposed, including introducing a target gene vector into an adeno-associated virus or lentivirus and delivering this to the patient (Axelsen and Woldbye, 2018). To actually apply this in humans, however, raises safety concerns, since it employs a virus. Cell therapy has emerged to compensate for the limitations of other treatments. In 1989, Olson’s group performed the first cell transplantation for PD patients (Lindvall et al., 1989). Ventral mesencephalic tissues derived from aborted human fetuses were transplanted into the striata of two patients, leading to improvement of certain motor symptoms. However, ethical problems may arise regarding the acquisition of fetal tissue, and even if ventral mesencephalic tissue is acquired, problems regarding purity control remain (Spenger et al., 1996).

Since then, a method for differentiating and transplanting cells derived from human embryonic stem cells (hESC) has been developed, and attempts have been made to overcome the limits of previous therapeutic agents. Particularly, after the discovery of dual-SMAD inhibition method in 2009 (Chambers et al., 2009), diverse and efficient differentiation protocols for DA neurons have been established. Once the techniques for generating human induced pluripotent stem cells (hiPSCs), another subset of hPSCs, from somatic cells were established (Takahashi et al., 2007), these hPSCs were subsequently directed towards differentiation into specific target cells, serving their purpose in cell therapy. Using the patient’s own cells (patient-derived hiPSCs) do not face ethical issues and do not result in immune reactions, as compared to those using embryonic-derived hESCs. Several groups have attempted using hPSC-based cell therapy for the fundamental treatment of PD. Various groups are attempting to generate ventral midbrain DA (vmDA) neurons derived from hPSCs, and confirming whether these neurons are indeed the intended vmDA neurons is crucial. Transplanting hPSC-derived vmDA progenitor cells without sufficient cell lineage quality control (QC) may result in the development of neoplastic tumor masses (Roy et al., 2006), as these cells could be contaminated with undifferentiated hPSCs and neural progenitor cells (NPCs) that have proliferative and differentiation capabilities. Therefore, precise QC is essential before transplantation. Ensuring a thorough QC process is crucial to minimize potential side effects that may occur after transplantation and to achieve appropriate clinical outcomes through cell therapy in PD. It is important to note that the current cell lineage QC methods fall short compared to the vmDA neuron differentiation protocol currently available. In this review, we intend to encompass the various types of PD therapy developed to date and analyze PD cell therapy research, along with an overview of vmDA neuron differentiation protocols and transplantation methods. Additionally, we assess the outcomes of *in vivo* experiments where differentiated cells are transplanted into a PD animal model, and we evaluate the resultant behavioral effects.

## 2 Conventional approaches to PD treatment

### 2.1 Medication

In the early 1960s, Birkmayer and Hornykiewicz were the first to report that levodopa, the precursor to dopamine, was an effective drug for treating PD (Birkmayer and Hornykiewicz, 1962, Birkmayer and Hornykiewicz, 1964). Since then, several medications have been developed through research aimed at understanding and addressing the neurodegenerative processes and symptoms of PD (Cheong et al., 2019). Levodopa, MAO-B inhibitors, *etc.*, are used to treat motor symptoms, which are the most apparent PD symptoms. Levodopa is a DA medication that helps to increase dopamine levels. When levodopa was administered to PD patients with low dopamine levels, their impaired motor function improved (Steiger et al., 1996). MAO-B inhibitors are non-DA drugs that may be substituted for powerful treatments, such as levodopa, in the early stages of PD (Ives et al., 2004). Psychosis, one of the non-motor symptoms of PD, is treated with pimavanserin, clozapine, and quetiapine (Seppi et al., 2019). Among the disadvantages of these various drug treatments are the decrease of drug effects over time, the possibility of drug-induced complications, and the occurrence of severe side effects, including motor fluctuations and dyskinesia, impulse-control disorder, sleepiness or sudden-onset sleep, and dopamine-dysregulation syndrome (Moore et al., 2014; Bastide et al., 2015). These medications can improve the symptoms of PD, but they cannot provide a fundamental cure.

### 2.2 Deep brain stimulation

In 1870, Fritsch and Hitzig demonstrated that they could generate movement by electrically stimulating the cerebral cortex in dogs (Fritsch and Hitzig, 2009). Afterwards, attempts were made to verify the function of the cerebral cortex and its inner structures *via* electrical stimulation. In 1960, it was reported for the first time that tremor could be reduced by stimulating the ventrolateral thalamus with a high frequency (Hassler et al., 1960). In 1987, stimulation of the ventral intermedius nucleus (VIM) improved tremor in drug-resistant PD patients (Benabid et al., 1987). DBS was approved by the U.S. Food and Drug Administration in 2002 and continues to be used to treat PD patients. Initially, many attempts were made to improve PD symptoms by stimulating the VIM of the thalamus. This method was able to reduce PD tremor but had no effect on other motor symptoms. Many PD patients are currently treated with electrodes implanted in the STN or the GPi. Both regions can reduce Parkinsonian tremor, but depending on the severity of non-motor symptoms, one region may be preferable over the other (Miocinovic et al., 2013). DBS may effectively improve symptoms in drug-resistant patients (Herrington et al., 2016), but surgical complications, such as hemorrhage or infection, and cost issues, such as expensive surgery costs and battery maintenance costs, may hamper its use (Bronstein et al., 2011).

### 2.3 Cell therapy using human fetal midbrain tissues

Transplantation of rodent ventral mesencephalic tissue into the 6-OHDA PD model marked the beginning of cell therapy for PD, as it was confirmed that this treatment improved symptoms in the apomorphine-induced rotation test (Bjorklund and Stenevi, 1979; Perlow et al., 1979). In the 1980s, clinical trials were initiated to transplant human fetal ventral mesencephalic tissue following the discovery of therapeutic effects in animal studies (Lindvall et al., 1988; Madrazo et al., 1988; Lindvall et al., 1989). Open-label trials demonstrated therapeutic effects in PD patients after fetal ventral mesencephalic tissue transplantation (Widner et al., 1992; Piccini et al., 1999). In the double-blind test, however, no significant impact was observed in the older patient group (Freed et al., 2001). Moreover, during the transplantation of fetal ventral mesencephalon that contained undifferentiated cells, the formation of neural rosettes was observed (Spenger et al., 1996). Additionally, the procedure of obtaining fetal ventral mesencephalon may raise ethical concerns. To overcome the limitations associated with fetal ventral mesencephalon transplantation for PD treatment, cell therapy has emerged by transplanting differentiated hPSCs into patients as DA progenitor cells (Table 1).

**TABLE 1:** Validation summary of dopaminergic neuronal differentiation from human pluripotent stem cells for transplantation.

hPSC line	Differentiation protocol (ref.)	Differentiation marker	Note	ID*	Ref
hpSC line LLC2P (hESC)	2D-based Gonzalez et al. (2013)	NESTIN, MUSASHI-1, SOX2 (ICC)	97.3% NESTIN^+^	a36	Gonzalez et al. (2016)
hpSC line LLC2P (hESC)	2D-based Gonzalez et al. (2013)	NESTIN, MUSASHI-1, SOX2 (ICC)	95.3% NESTIN^+^, 96.7% SOX2^+^	b45	Gonzalez et al. (2015)
RC-17 (hESC)	2D-based Nolbrant et al. (2017)	n/a	No *in vitro* data	b52	Hoban et al. (2020)
RC-17, WA09 (hESC)	2D-based Nolbrant et al. (2017)	n/a	No *in vitro* data	a16	Cardoso et al. (2018)
RC-17 (hESC)	2D-based Chambers et al. (2009)	FOXA2, LMX1A, LMX1B, OTX2 (ICC); *CORIN, EN1, FOXA2, LMX1A, LMX1B* (qRT-PCR)	Exhibited higher expression of FOXA2 and LMX1A compared to the forebrain patterned protocol	b41	Adler et al. (2019)
RC-17 (hESC)	2D-based Eiraku et al. (2008); Chambers et al. (2009); Fasano et al. (2010)	FOXA2, LMX1A, OTX2, TH (ICC); *FOXA2, LMX1A, OTX2* (qRT-PCR)	Used bimodal imaging to investigate the fate of vmDA progenitors	b53	Mousavinejad et al. (2020)
SA002.5 (hESC)	2D-based Kawasaki et al. (2000); Kawasaki et al. (2002)	DA (HPLC); TH, TUJ1 (ICC)	38 ± 22% TH^+^, 42 ± 20% TUJ1^+^	a13	Brederlau et al. (2006)
WA01 (hESC)	2D-based Chambers et al. (2009); Fasano et al. (2010)	FOXA2, OTX2, TH (ICC)	[DIV11] ∼83% FOXA2^+^, 81.5 ± 5.0% FOXA2^+^OTX2^+^, ∼91% OTX2^+^	a26	de Luzy et al. (2021)
			[DIV19] 5.6 ± 4.3% TH^+^, ∼9% TH^+^/FOXA2^+^		
			[DIV25] ∼77% FOXA2^+^, ∼87% OTX2^+^, 30.1 ± 3.6% TH^+^, ∼33% TH^+^/FOXA2^+^		
WA01, WA09 (hESC); 2C6, SeV6 (hiPSC)	2D-based Chambers et al. (2009); Fasano et al. (2010)	DA (HPLC); DAT, FOXA2, GIRK2, LMX1A, NURR1, OTX2, TH, TUJ1 (ICC); *ASCL1, FOXA2, LMX1A, NURR1, PITX3* (qRT-PCR)	[DIV11] ∼94% FOXA2^+^, ∼83% LMX1A^+^, ∼76% LMX1A + FOXA2^+^, ∼90% OTX2^+^, ∼80% OTX2^+^FOXA2^+^	a5	Kriks et al. (2011)
			[DIV25] ∼92% LMX1A^+^FOXA2^+^, ∼40% NURR1^+^FOXA2^+^, ∼18% TH^+^FOXA2^+^		
			[DIV50] ∼85% FOXA2^+^, ∼76% LMX1A^+^, ∼58% NURR1^+^, ∼80% TH^+^		
WA07, WA09 (hESC)	2D-based Perrier et al. (2004)	OTX2, PAX2, TH, TUJ1 (ICC); *TH, TUJ1* (qRT-PCR); *EN1, LMX1A, MSX1, NURR1, PAX2, PITX3, TH* (RT-PCR)	[DIV37] 10.8% TH^+^ (1-week Noggin); 23.6% TH^+^ (3-week Noggin)	a12	Sonntag et al. (2007)
			[DIV49] 8.6% TH+ (1-week Noggin); 22.3% TH^+^ (3-week Noggin)		
WA07, WA09 (hESC); C4 (hiPSC)	2D-based Chambers et al. (2009); Fasano et al. (2010)	FOXA2, LMX1A, NURR1, TH (ICC); *FOXA2, LMX1A, TH* (qRT-PCR)	[DIV26, clinical run 1] ∼100% FOXA2^+^, ∼97% LMX1A^+^, ∼97% FOXA2^+^/LMX1A^+^, ∼37% NURR1^+^, ∼24% TH^+^	b43	Schweitzer et al. (2020)
			[DIV26, clinical run 2] ∼96% FOXA2^+^, ∼70% LMX1A^+^, ∼68% FOXA2^+^/LMX1A^+^, ∼10% NURR1^+^, ∼15% TH^+^		
WA09 (hESC)	2D-based Chambers et al. (2009); Fasano et al. (2010)	FOXA2, GFP [ = PITX3 or LMX1A], NURR1, OTX2, TH (ICC); *LMX1A, TH* (qRT-PCR)	[DIV15] Checked FOXA2^+^OTX2^+^ or GFP [ = LMX1A]^+^FOXA2^+^OTX2^+^ vmDA progenitors	a29	Gantner et al. (2020)
			[DIV21] Checked GFP [ = LMX1A]^+^NURR1^+^TH^+^ vmDA neurons		
			[DIV25] Checked GFP [ = PITX3]^+^TH^+^FOXA2^+^ vmDA neurons		
WA09 (hESC)	2D-based Chambers et al. (2009); Fasano et al. (2010)	FOXA2, LMX1A, MAP2, NURR1, TH, TUJ1 (ICC)	[DIV20] ∼92% LMX1A^+^FOXA2^+^	a3	Steinbeck et al. (2015)
			[DIV30] ∼53% NURR1^+^		
WA09 (hESC)	2D-based Eiraku et al. (2008); Chambers et al. (2009); Fasano et al. (2010)	FOXA2 (FACS); FOXA2, TH (ICC); *ASCL1, CORIN, DDC, EN1, FOXA2, LMX1A/B, NURR1, OTX2, PITX3, TH* (qRT-PCR)	Tested with large-scale manufactured cryopreserved clinical-grade vmDA progenitor stocks (MSK-DA01)	a25	Piao et al. (2021)
WA09 (hESC); A6, PDA^3F^-1, PDC^3F^-1, PDB^3F^-17Puro-5, PDB^3F^-21Puro-26 (hiPSC)	2D-based Okabe et al. (1996); Lee et al. (2000)	TH, TUJ1(ICC)	[DIV42, WA09] ∼5% TH^+^, ∼20% TUJ1^+^, ∼34% TH^+^/TUJ1^+^	a10	Hargus et al. (2010)
			[DIV42, A6] ∼6% TH^+^, ∼20% TUJ1^+^, ∼44% TH^+^/TUJ1^+^		
			[DIV42, PDA^3F^-1] ∼ 3% TH^+^, ∼22% TUJ1^+^, ∼19% TH^+^/TUJ1^+^		
			[DIV42, PDC^3F^-1] ∼10% TH^+^, ∼30% TUJ1^+^, ∼39% TH^+^/TUJ1^+^		
			[DIV42, PDB^3F^-17Puro-5] ∼6% TH^+^, ∼20% TUJ1^+^,∼ 29% TH^+^/TUJ1^+^		
			[DIV42, PDB^3F^-21Puro-26] ∼6% TH^+^, ∼20% TUJ1^+^, ∼41% TH^+^/TUJ1^+^		
WA09 (hESC); C4 (hiPSC)	2D-based Chambers et al. (2009); Fasano et al. (2010)	DA (HPLC); ALDH1A1, DAT, GIRK2, FOXA2, LMX1A, NURR1, MAP2, PITX3, TH, VMAT2 (ICC); *CORIN, DAT, EN1, FOXA2, GIRK2, LMX1A, NURR1, OTX2, PITX3, TH* (qRT-PCR)	[DIV28] >80% FOXA2^+^LMX1A^+^, ∼40% MAP2^+^, ∼38% NURR1^+^, ∼ 15% TH^+^; majority of TH^+^ cells co-expressed FOXA2, LMX1A, and NURR1	a27	Song et al. (2020)
WA09 (hESC); Epi-iPS, pt-hiPSC (hiPSC)	2D-based Chambers et al. (2009); Fasano et al. (2010)	DA (ELISA); EN1, FOXA2, LMX1A, MAP2, NURR1, OTX2, TH, TUJ1, VMAT2 (ICC); *FOXA2, LMX1A, MASH1, NURR1, OTX2* (qRT-PCR)	[LIN28A^p.R192G/+^ PD hiPSC] ∼28% TH^+^	a22	Chang et al. (2019)
			[LIN28A^corrected/+^ PD hiPSC] ∼48% TH^+^		
WA09, HES-3 (hESC); RM3.5, 409B-2 (hiPSC)	2D-based Chambers et al. (2009); Fasano et al. (2010)	DA (HPLC); FOXA2, GFP [ = LMX1A or PITX3], NURR1, OTX2, TH (ICC); *FOXA2, LMX1A, NURR1, PITX3, TH* (qRT-PCR)	[Xenogeneic condition] 72.2% FOXA2^+^, 87.8% OTX2^+^, 70.0% OTX2^+^FOXA2^+^ (WA09); 76.5% FOXA2^+^, 77.9% OTX2^+^, 69.6% OTX2^+^FOXA2^+^ (RM3.5)	a21	Niclis et al. (2017)
			[Xenogeneic-free condition] 89.0% FOXA2^+^, 48.6% FOXA2^+^TH^+^, 96.8% OTX2^+^, 88.9% OTX2^+^FOXA2^+^, 50.1% TH^+^, (WA09); 52.9% FOXA2^+^TH^+^, 91.3% OTX2^+^FOXA2^+^, 55.7% TH^+^ (HES-3); 85.6% FOXA2^+^, 67.9% FOXA2^+^TH^+^, 88.9% OTX2^+^, 82.9% OTX2^+^FOXA2^+^, 76.2% TH^+^ (RM3.5); 90.3% OTX2^+^FOXA2^+^ (409B-2)		
WA09, MEL-1 (hESC); MRC-5 (hiPSC)	2D-based Eiraku et al. (2008); Chambers et al. (2009); Fasano et al. (2010)	DA (HPLC); EN1, FOXA2, LMX1A, MAP2, NURR1, OTX2, TH (ICC); *ADCYAP1, CHIRNA4, EN1, FOXA2, GIRK2, LMX1A, NURR1, OTX2, PITX3, SNCA* (qRT-PCR)	[0.7 µM CHIR99021] ∼0% EN1^+^, ∼95% FOXA2^+^	a24	Kim et al. (2021b)
			[0.7➚7.5 µM CHIR99021] ∼90% EN1^+^, ∼99% FOXA2^+^		
WA09, UC-06 (hESC); IMR90-1, IMR90-4, Foreskin-1, SES8, Rv-hiPS01-1, Rv-hiPS02-3, piPSC-#1, piPSC-#2 (hiPSC)	2D-based Okabe et al. (1996); Kawasaki et al. (2000); Lee et al. (2000); Perrier et al. (2004)	DA (HPLC); DAT, GIRK2, NURR1, TH, TUJ1 (ICC); *AADC, DAT, EN1, GIRK2, LMX1A, LMX1B, NURR1, VMAT2* (RT-PCR)	38.38 ± 2.54% TH^+^/TUJ1^+^ (passage 2); ∼43% TH^+^/TUJ1^+^ (passage 4); ∼48% TH^+^TUJ1^+^ (passage 6); 43.37 ± 3.52% TH^+^TUJ1^+^ (passage 8)	a19	Rhee et al. (2011)
DF6-9, DF19-9 (hiPSC)	2D-based Chambers et al. (2009); Fasano et al. (2010)	DA (ELISA); FOXA2, MAP2, TH (FACS); FOXA2, LMX1A, MAP2, TH (ICC); *AADC, DRD2, EN1, FOXA2, GIRK2, LMX1A, NURR1, OTX2, PITX3, TH, VMAT2* (qRT-PCR); EN1, FOXA2, GIRK2, MAP2, PITX3, TH, VMAT2 (WB)	[7 days after thawing cryopreserved vmDA neuron stocks] 91% FOXA2^+^LMX1A^+^	a15	Wakeman et al. (2017)
			[14 days after thawing cryopreserved vmDA neuron stocks] 72.3% FOXA2^+^TH^+^, 97.0% MAP2^+^		
healthy hiPSC, PD hiPSC (hiPSC)	2D-based Chambers et al. (2009); Fasano et al. (2010)	FOXA2, LMX1A, TH, TUJ1 (ICC); *FOXA2, MAP2, NURR1, TH* (qRT-PCR)	[DIV11, healthy hiPSC] 44.01 ± 5.67% FOXA2^+^LMX1A^+^	b44	Zygogianni et al. (2019)
			[DIV11, SNCA^p.A53T/+^ PD hiPSC] 48.63 ± 7.32% FOXA2^+^LMX1A^+^		
			[DIV30, healthy hiPSC] 10.85 ± 0.97% TH^+^, 67.35 ± 9.41% TUJ1^+^		
			[DIV30, SNCA^p.A53T/+^ PD hiPSC] 9.86 ± 2.84% TH^+^, 63.29 ± 14.19% TUJ1^+^		
PD-1, PD-2, NCF-1, NCF-2, NCF-3 (hiPSC)	2D-based Zhang et al. (2001); Perrier et al. (2004); Yan et al. (2005)	TH, TUJ1 (ICC)	[DIV35] 76% NESTIN^+^, 33% TH^+^, 71% TUJ1^+^	a8	Han et al. (2015)
TZ16 (hiPSC)	2D-based Chambers et al. (2009); Fasano et al. (2010)	DA (ELISA); FOXA2, TH, TUJ1 (ICC); *AADC, DRD2, EN1, FOXA2, GIRK2, LMX1A, NURR1, OTX2, PTX3, TH, VMAT2* (RNA-seq)	Checked FOXA2^+^TH^+^ and TH^+^TUJ1^+^ cells at DIV23 or DIV50	a2	Leitner et al. (2019)
BG01, BG03 (hESC)	EB-based Schulz et al. (2003)	DA (HPLC); AADC, DAT, MAP2, TH, TUJ1, VMAT2 (ICC); *AADC, DAT, EN1, GIRK2, LMX1B, MAP2, NURR1, PITX3, TH, VMAT2* (RT-PCR)	63.8 ± 4.6% TH^+^/MAP2^+^, 73.9 ± 10.5% TH^+^/TUJ1^+^, 94.9 ± 2.9% VMAT2^+^/MAP2^+^	b48	Schulz et al. (2004)
ES [2], ES [4] (hESC); KIPS-4F, FIPS-FA (hiPSC)	EB-based Kawasaki et al. (2000)	DA (ELISA); DAT, FOXA2, GIRK2, LMX1A, TH, TUJ1 (ICC); *ALDH1A1, EN1, LMX1A, NURR1, TH* (qRT-PCR)	∼50% TUJ1^+^, ∼53% TH^+^/TUJ1^+^	b42	Sanchez-Danes et al. (2012)
hES1 (hESC)	EB-based Lee et al. (2000)	MAP2, TH, TUJ1, (ICC); DA (RP-HPLC); *AADC, DAT, EN1, LMX1b, NURR1, TH* (RT-PCR)	40% TH^+^/TUJ1^+^	a11	Geeta et al. (2008)
KhES-1 (hESC)	EB-based Eiraku et al. (2008); Chambers et al. (2009); Fasano et al. (2010)	FOXA2, LMX1A, NURR1, TH, TUJ1 (ICC); *CORIN, FOXA2, MAP2ab, NURR1, TH* (qRT-PCR)	[DIV24] ∼50% FOXA2^+^LMX1A^+^	a35	Samata et al. (2015)
			[DIV35] ∼100% TUJ1^+^, some were TH^+^/TUJ1^+^, most TH^+^ cells were also FOXA2^+^ and NURR1^+^		
SNUhES1, SNUhES3, SNUhES16 (hESC)	EB-based (n/a)	TH, TUJ1 (FACS); DA (HPLC); AADC, EN1, TH, TUJ1 (ICC); *EN1, NURR1, PITX3* (RT-PCR)	[SNUhES1] 91.61 ± 0.64% EN1^+^/TH^+^, 86 ± 1.4% TH^+^/TUJ1^+^, 77% TUJ1^+^, most TH^+^ cells expressed AADC	a9	Cho et al. (2008)
			[SNUhES3] 77.18 ± 1.36% TH^+^/TUJ1^+^		
			[SNUhES16] 81.74 ± 1.52% TH^+^/TUJ1^+^		
unclear (hESC)	EB-based Zhang et al. (2001); Yan et al. (2005); Chambers et al. (2009); Fasano et al. (2010)	FOXA2, LMX1A, MSX1, TH, TUJ1 (ICC); *DAT, EN1, FOXA2, GIRK2, LMX1A, NURR1, OTX2, PITX3, TH, TUJ1* (qRT-PCR)	[DIV25] Checked FOXA2^+^LMX1A^+^ cells	b50	Adil et al. (2017)
			[DIV40] ∼70-90% FOXA2^+^LMX1A^+^		
WA01 (hESC)	EB-based Reynolds and Weiss (1992); Lee et al. (2000)	DA (ELISA); NURR1, TH, TUJ1 (ICC); *DAT, NURR1, TH, TUJ1* (qRT-PCR)	17.13 ± 1.59% TH^+^, 95.68 ± 0.92% TUJ1^+^	b55	Wakeman et al. (2014)
WA01, WA09 (hESC); 1,588, 27,760, HUF1, HUF6 (hiPSC)	EB-based Chambers et al. (2009); Fasano et al. (2010)	RFP [ = TH] (FACS); DA (HPLC); AADC, FOXA2, GIRK2, LMX1A, NURR1, OTX2, TH, TUJ1 (ICC); *EN1, FOXA2, LMX1A, NURR1, TH* (qRT-PCR)	[DIV25] ∼14% TH^+^	b51	Xia et al. (2017)
			[DIV35] ∼24% TH^+^		
			[DIV50] ∼35% TH^+^		
WA09 (hESC)	EB-based Ying et al. (2003); Watanabe et al. (2005); Chambers et al. (2009)	FOXA2, LMX1A, OTX2 (ICC)	Checked FOXA2^+^LMX1A^+^ and OTX2^+^LMX1A^+^ cells	a20	Grealish et al. (2015)
WA09 (hESC)	EB-based Perrier et al. (2004); Chambers et al. (2009); Fasano et al. (2010)	FOXA2, LMX1A, MAP2, NURR1, OTX2, TH (ICC)	[DIV16] Checked FOXA2^+^LXM1A^+^ and OTX2^+^ cells	a17	Grealish et al. (2014)
WA09, RC-17, HS980a (hESC); Miltenyi iPSCs (hiPSC)	EB-based Perrier et al. (2004); Fasano et al. (2010)	*DDC, DLK1, EN1, FOXA2, LMX1A, NURR1, OTX2, PBX1, PITX3, TH* (scRNA-seq)	Analyzed cells *via* scRNA-seq pre-grafting and 6 months post-transplantation	a28	Tiklova et al. (2020)
WA09, RoyanH6 (hESC)	EB-based Chambers et al. (2009)	GFP [ = LMX1A^GFP/+^] (FACS); CORIN, FOXA2, GIRK2, LMX1A, MAP2, OTX2, PITX3, TH (ICC); *EN1, FOXA2, LMX1A, LMX1B, MSX1, PITX3* (qRT-PCR)	[DIV12] ∼53% CORIN^+^, ∼68% FOXA2^+^, ∼46% LMX1A^+^, ∼58% OTX2^+^ (unsorted); 63 ± 6.7% CORIN^+^, 91 ± 3.9% FOXA2^+^, 84 ± 4.7% LMX1A^+^, 93 ± 5.3% OTX2^+^ (GFP [ = LMX1A^GFP/+^]^+^ sorted)	a30	Fathi et al. (2018)
			[DIV30] 82 ± 8.7% TH^+^/GIRK2^+^, 89 ± 5.4% TH^+^/MAP2^+^, 82 ± 10% TH^+^/PITX3^+^ (GFP [ = LMX1A^GFP/+^]^+^ sorted)		
IMR90 (hiPSC)	EB-based Iacovitti et al. (2007)	DA (HPLC); ALDH1A1, LMX1A, TH, TRKB (ICC); *ALDH1A1, FOXA2, LMX1A, MSX1, NURR1, PITX3, TH* (RT-PCR)	6.5 ± 1.4% TH^+^	a18	Cai et al. (2010)
K2 (hiPSC)	EB-based Eiraku et al. (2008)	TUJ1, TH (ICC)	[DIV46, protocol 1] 3.5 ± 0.8% TH^+^, 42 ± 3.8% TUJ1^+^	b46	Effenberg et al. (2015)
			[DIV46, protocol 2] 7.4 ± 1.2% TH^+^, 49.3 ± 3% TUJ1^+^		
MR31, MMW2 (hiPSC)	EB-based Swistowski et al. (2009)	GIRK2, TH, TUJ1 (ICC); *AADC, DAT, EN1, GIRK2, LMX1B, MSX1, NURR1, OTX2, TH, VMAT* (qRT-PCR)	∼100% GIRK2^+^/TH^+^, 30 ± 5% TH^+^	a6	Swistowski et al. (2010)
ES01 (hESC)	neurosphere-based Ben-Hur et al. (2004)	TH, TUJ1 (ICC); AADC, *EN1, EN2, LMX1B, NURR1, OTX2, PAX2, PAX5, PTX3, TH* (RT-PCR)	29 ± 0.6% TUJ1^+^, 0.56 ± 0.05% TH^+^TUJ1^+^	a14	Ben-Hur et al. (2004)
HES-3 (hESC)	neurosphere-based Eiraku et al. (2008); Chambers et al. (2009); Fasano et al. (2010)	DA (HPLC); TH, TUJ1 (ICC); DAT, TH (WB)	Evaluated DAT and TH expression levels *via* WB, observing DAT expression starting at DIV12 and TH expression starting at DIV24	a23	Goggi et al. (2020)
KhES-1 (hESC); 1039A-1 (hiPSC)	neurosphere-based Eiraku et al. (2008)	DA (HPLC); DAT, FOXA2, LMX1A, NURR1, PITX3, TH, TUJ1 (ICC)	[DIV14, hESC] 75.9 ± 4.5% FOXA2^+^LMX1A^+^ (unsorted); 89.5 ± 1.5% FOXA2^+^LMX1A^+^ (LRTM1^+^ sorted)	a31	Samata et al. (2016)
			[DIV14, hiPSC] 72.0 ± 1.3% FOXA2^+^LMX1A^+^ (unsorted); 86.7 ± 2.6% FOXA2^+^LMX1A^+^ (LRTM1^+^ sorted)		
			[DIV28, hESC] ∼7% TH^+^FOXA2^+^, ∼11% TH^+^NURR1^+^, ∼82% TUJ1^+^ (unsorted); ∼39% TH^+^FOXA2^+^, ∼38% TH^+^NURR1^+^, ∼94% TUJ1^+^(LRTM1^+^ sorted)		
			[DIV28, hiPSC] ∼7% TH^+^FOXA2^+^, ∼6% TH^+^NURR1^+^, ∼77% TUJ1^+^ (unsorted); ∼37% TH^+^FOXA2^+^, ∼34% TH^+^NURR1^+^, ∼91% TUJ1^+^ (LRTM1^+^ sorted)		
KhES-1, KhES-2 (hESC)	neurosphere-based Kawasaki et al. (2000); Kawasaki et al. (2002)	DA (HPLC); AADC, FOXA2, GIRK2, NURR1, OTX2, PITX3, TH (ICC); *CORIN, EN1, FOXA2, LMX1A, NURR1, TH* (qRT-PCR)	[DIV28] 11.7 ± 2.2% TH^+^/TUJ1^+^	a38	Doi et al. (2012)
			[DIV35] 25.1 ± 6.6% TH^+^/TUJ1^+^		
			[DIV42] 34.7 ± 7.3% TH^+^/TUJ1^+^		
			[DIV56] 44.7 ± 6.6% NURR1^+^/TH^+^		
WA09 (hESC)	neurosphere-based Amit et al. (2003)	DAT, EN1, GIRK2, MAP2, TH, VMAT2 (ICC); *EN1, GIRK2, MAP2, NURR1, TH* (qRT-PCR)	[Neural stage 1] Lenti-MEF2CA-infected cells showed a 2.4-fold increase in TH^+^ neurons compared to control-infected cells	a7	Cho et al. (2011)
			[Neural stage 2] Lenti-MEF2CA-infected cells exhibited ∼4-fold more EN^+^ cells compared to control-infected cells		
			[Neural stage 3] 54.6 ± 3.0% TH^+^/MAP2^+^		
WA09 (hESC)	neurosphere-based Chambers et al. (2009)	EN1, FOXA2, GIRK2, LMX1A, NURR1, OTX2, TH, TUJ1 (ICC)	[DIV32] ∼88% CORIN^+^, ∼99% EN1^+^, ∼98% EN1^+^/TH^+^, ∼98% FOXA2^+^, ∼98% FOXA2^+^/TH^+^, ∼88% GIRK2^+^/TH^+^, ∼98% LMX1A^+^, ∼97% LMX1A^+^/TH^+^, ∼98% NURR1^+^/TH^+^, ∼70% TH^+^, ∼86% TH^+^/TUJ1^+^	a4	Xiong et al. (2021)
201B-7, 1147F-1 (hiPSC)	neurosphere-based Eiraku et al. (2008); Chambers et al. (2009); Fasano et al. (2010)	FOXA2, NURR1, TH (ICC); *CORIN* (qRT-PCR)	[DIV19] ∼82% FOXA2^+^/KI67^+^	b47	Katsukawa et al. (2016)
201B-7, 253G-1 (hiPSC)	neurosphere-based Chambers et al. (2009); Fasano et al. (2010)	DA (HPLC); TH, TUJ1 (ICC)	[DIV48] 63 ± 13% TH^+^, >50% TH^+^/TUJ1^+^	b49	Komatsu et al. (2015)
253G-4 (hiPSC)	neurosphere-based Eiraku et al. (2008)	DA (HPLC); TH, TUJ1 (ICC); *TH* (qRT-PCR)	[DIV28] 3.14 ± 1.38% TH^+^/TUJ1^+^, most cells were TUJ1^+^	a37	Kikuchi et al. (2011)
			[DIV42] 85.46 ± 3.13% TH^+^/TUJ1^+^		
253G-4, 404C-2 (hiPSC)	neurosphere-based Eiraku et al. (2008)	MAP2, TH, TUJ1 (ICC); *MAP2ab*, *TH* (qRT-PCR)	[DIV22] 3.3 ± 1.9% MAP2ab^+^, 9.0 ± 3.3% TH^+^/TUJ1^+^, ∼54% TUJ1^+^ (control); 57.7 ± 8.3% MAP2ab^+^, 12.8 ± 4.0% TH^+^/TUJ1^+^, ∼77% TUJ1^+^ (DAPT-treated condition); 72.7 ± 5.6% MAP2ab^+^, 13.1 ± 2.8% TH^+^/TUJ1^+^, ∼85% TUJ1^+^ (compound E-treated condition)	b40	Ogura et al. (2013)
			[DIV25] 203.2 ± 24.2 μm (control), 384.4 ± 37.2 μm (DAPT-treated condition), or 496.2 ± 48.5 μm (compound E-treated condition) of TH^+^ neurites length		
404C-2, 836B-3 (hiPSC)	neurosphere-based Eiraku et al. (2008); Chambers et al. (2009); Fasano et al. (2010)	DA (HPLC); AADC, CORIN, FOXA2, GIRK2, NURR1, PITX3, TH (ICC); *CORIN, EN1, FOXA2, LMX1A, NURR1, OTX2, PITX3, TH* (qRT-PCR)	[DIV12] 18.9 ± 15.4% CORIN^+^, 47.3 ± 6.6% LMX1A^+^FOXA2^+^ (unsorted); 75.5 ± 8.2% LMX1A^+^FOXA2^+^ (CORIN^+^ sorted); ∼36% LMX1A^+^FOXA2^+^ (CORIN^−^ sorted)	a32	Doi et al. (2014)
			[DIV21] 45.4 ± 14.6% CORIN^+^		
			[DIV28] ∼63% FOXA2^+^, ∼20% NURR1^+^, ∼10% TH^+^ (unsorted); ∼75% FOXA2^+^, 27.3 ± 5.5% NURR1^+^, 2.1 ± 1.2% TH^+^ (CORIN^+^ sorted)		
			[DIV42] ∼35% FOXA2^+^, ∼32% NURR1^+^, ∼20% TH^+^ (unsorted); ∼70% FOXA2^+^, 19.9 ± 6.9% NURR1^+^, 42 ± 4.4% TH^+^ (CORIN^+^ sorted)		
783E-2, 836B-3, 1147F-1, 1231A-3, 1263A-18, 1274A-8, 1274A-15, 1275A-2, 1275A-3, N112-4, N117-8, PD-17-7 (hiPSC)	neurosphere-based Eiraku et al. (2008); Chambers et al. (2009); Fasano et al. (2010)	DA (HPLC); NURR1, FOXA2, NURR1 (ICC)	[DIV26, CORIN^+^ sorted] 95.3 ± 1.6% FOXA2^+^, 17.8 ± 2.4% NURR1^+^ (healthy hiPSC); 97.2 ± 2.2% FOXA2^+^, 15 ± 0.8% NURR1^+^ (PD hiPSC)	a34	Kikuchi et al. (2017b)
783E-2, 836B-3, 1147F-1, 1231A-3, 1263A-18, 1275A-3, N117-11, PD12-1 (hiPSC)	neurosphere-based Eiraku et al. (2008)	CORIN (FACS); DA (HPLC); FOXA2, NURR1, TUJ1 (ICC)	[DIV12] 12.5% CORIN^+^ (N117-11); 15.5% CORIN^+^ (1147F1); 25.5% CORIN^+^ (836B3); 25.9% CORIN^+^ (1231A3); 26.9% CORIN^+^ (PD12-1); 21.6% CORIN^+^ (783E2); 15.6% CORIN^+^ (1275A3); 15.4% CORIN^+^ (1263A18)	a39	Kikuchi et al. (2017a)
			[DIV26, CORIN^+^ sorted] 94.2% FOXA2^+^, 87% FOXA2^+^TUJ1^+^, 15% NURR1^+^ (N117-11); 91.5% FOXA2^+^, 90.6% FOXA2^+^TUJ1^+^, 12.6% NURR1^+^ (1147F1); 98.9% FOXA2^+^, 88.9% FOXA2^+^TUJ1^+^, 22.8% NURR1^+^ (836B3); 96.9% FOXA2^+^, 90.6% FOXA2^+^TUJ1^+^, 20.9% NURR1^+^ (1231A3); 99% FOXA2^+^, 88% FOXA2^+^TUJ1^+^, 17.3% NURR1^+^ (PD12-1); 88.3% FOXA2^+^, 83.1% FOXA2^+^TUJ1^+^, 12.1% NURR1^+^ (783E2); 99.6% FOXA2^+^, 85.2% FOXA2^+^TUJ1^+^, 14.4% NURR1^+^ (1275A3); 99.7% FOXA2^+^, 86.9% FOXA2^+^TUJ1^+^, 16% NURR1^+^ (1263A18)		
1039A-1 (hiPSC)	neurosphere-based Eiraku et al. (2008); Chambers et al. (2009); Fasano et al. (2010)	FOXA2, NURR1, TH (ICC)	[DIV28] Checked NURR1^+^FOXA2^+^ cells	a33	Nishimura et al. (2016)
			[DIV56] Checked FOXA2^+^TH^+^ cells		
1039A-1 (hiPSC)	neurosphere-based Eiraku et al. (2008); Chambers et al. (2009); Fasano et al. (2010)	FOXA2, NURR1, TH (ICC)	[DIV28] 92.3 ± 2.1% FOXA2^+^, 48.3 ± 4.0% NURR1^+^, 6.0 ± 1.6% TH^+^/NURR1^+^	b54	Miyawaki et al. (2020)
HFF-1 (hiPSC)	neurosphere-based Kawasaki et al. (2000); Kawasaki et al. (2002); Perrier et al. (2004); Vazin et al. (2009); Schwartz et al. (2012)	EN1, FOXA2, MAP2, PITX3, TH, TUJ1 (ICC); *DDC, EN1, FOXA2, LMX1B, MAP2, NURR1, OTX2, TH* (qRT-PCR)	[DIV22] 94.1 ± 0.76% MAP2^+^, 92.5 ± 1.73% TH^+^ (2D-based); 93.6 ± 1.53% MAP2^+^, 92.3 + 1.52% TH^+^ (neurosphere-based)	a1	Francis et al. (2020)

* Papers with IDs a1 to a39 assessed the impact of cell transplantation on motor function recovery, while papers with IDs b40 to b55 did not assess the effects of motor function recovery after cell transplantation.

** 2D, monolayer differentiation. DA, dopamine; DIV, days *in vitro*; ELISA, confirmed by enzyme-linked immunosorbent assay; EB, embryonic body differentiation; hESC, human embryonic stem cell; hiPSC, human induced pluripotent stem cell; HPLC, confirmed by high performance liquid chromatography; hpSC, human parthenogenetic stem cell; ICC, confirmed by immunocytochemistry. n/a, not available. neurosphere, neurosphere differentiation. PD, Parkinson’s disease. qRT-PCR, confirmed by quantitative real-time PCR. RNA-seq, confirmed by RNA, sequencing; RP-HPLC, confirmed by reverse-phase high performance liquid chromatography; RT-PCR, confirmed by reverse transcription PCR., scRNA-seq, confirmed by single cell RNA, sequencing; vmDA, ventral midbrain dopaminergic; WB, confirmed by western blotting.

## 3 Advances in hPSC-derived DA progenitor transplantation research for PD cell therapy: A comprehensive review of preclinical studies

### 3.1 Literature search strategy

A comprehensive literature search was conducted using PubMed to identify relevant papers published until 31 October 2021. The search terms used were as follows: (Parkinson) AND (hiPSC OR hESC) AND (transplantation). In total, 329 articles were retrieved from the search. To assess the relevance of each publication, the titles, keywords, and abstracts were evaluated. From the initial pool, 2 duplicate articles, 2 non-English articles, 170 articles categorized as reviews, correspondences, or editorials, and 91 articles that did not involve the actual use of hESC or hiPSC in transplantation were excluded, leaving 55 articles eligible for analysis. These 55 papers were selected and reviewed, focusing on their reports regarding the outcomes of cell transplantation using hPSC-derived neuronal cells.

### 3.2 Evolution and specialization of hPSC-derived DA progenitors for PD cell therapy

DA neurons derived from hESCs are believed to have the potential to replace the degenerated DA neurons in the PD brain (Ben-Hur et al., 2004). These neurons utilize dopamine as a neurotransmitter and express tyrosine hydroxylase (TH), the rate-limiting enzyme in dopamine synthesis (Kosaka et al., 1987). While TH-expressing DA neurons are found in multiple brain regions, those primarily associated with PD are located in the ventral midbrain. Specifically, vmDA neurons are distributed across three regions: the SNpc (A9), the ventral tegmental area (VTA, A10), and the retrorubral field (RrF, A8) (Hokfelt et al., 1974). vmDA neurons originating from each of these regions project to distinct locations (Lammel et al., 2008). Age-related loss of vmDA neurons in the SNpc, affecting the nigrostriatal pathway from A9 to the dorsal striatum, is the underlying cause of PD (Kish et al., 1988; Fearnley and Lees, 1991). To address this vmDA neuronal loss in the SNpc, several research groups have developed hESC-based differentiation protocols for generating DA neurons, aiming to utilize them in cell therapy approaches (Table 1). Initially, experiments using mouse ESCs were conducted (Kawasaki et al., 2000), followed by hESC experiments focusing on the differentiation of vmDA neurons (Perrier et al., 2004). More recently, with the advancement of iPSCs (Takahashi and Yamanaka, 2006; Takahashi et al., 2007), differentiation experiments using patient-derived iPSCs to generate vmDA neurons have been gradually progressing. The vmDA neurons differentiated from hPSCs originate from the ventral midbrain floor plate (vmFP), which is the most ventral region of the neural tube. Co-expression of the floor plate marker Forkhead box protein A2 (FOXA2) and the roof plate marker LIM homeobox transcription factor 1 alpha (LMX1A) generally identifies the progenitor cells in this region, known as vmDA progenitors (Andersson et al., 2006; Bonilla et al., 2008).

### 3.3 Advances and strategies in direct differentiation of PSCs into DA neurons

**FIGURE 1:**
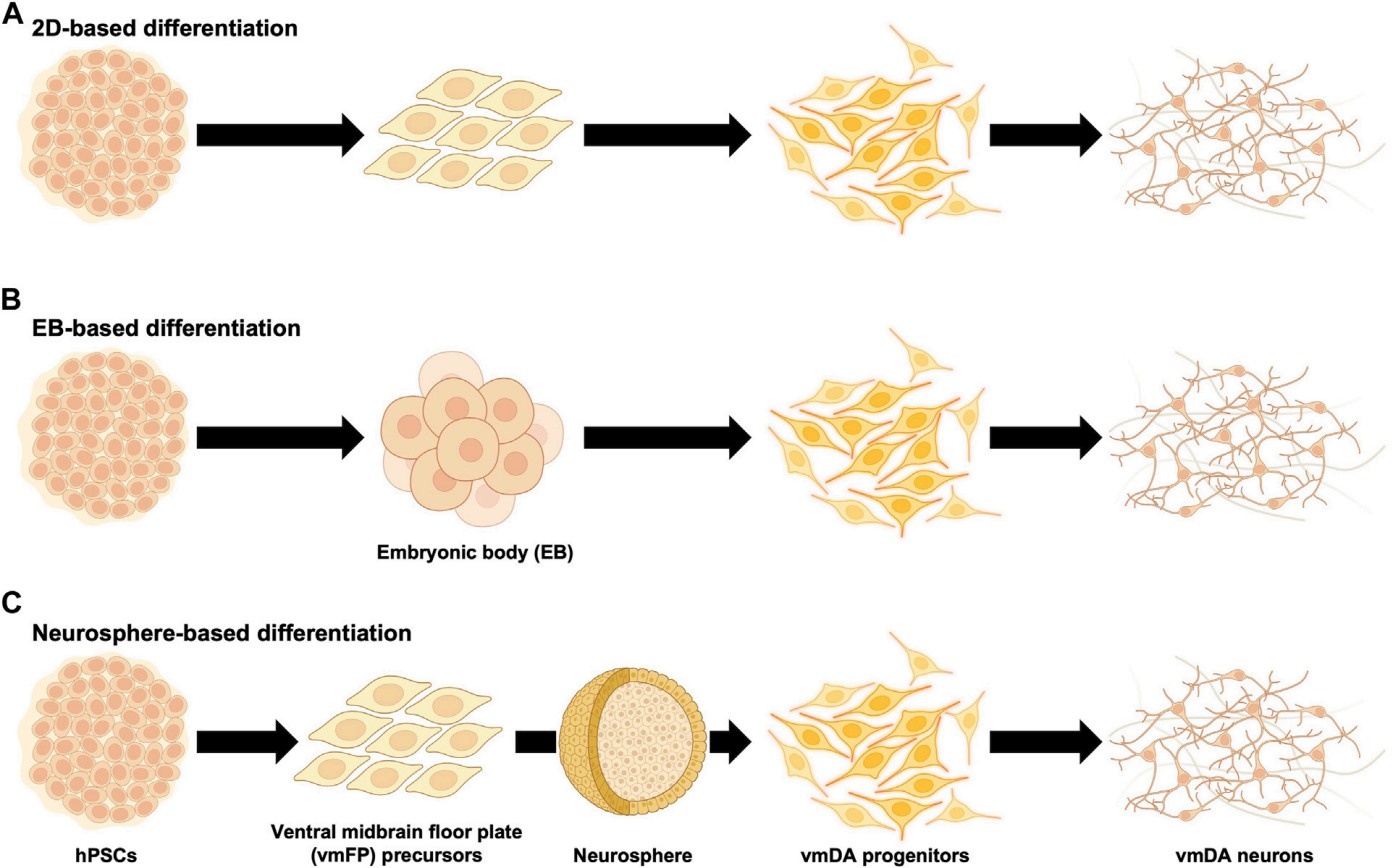
Diagram summarizing differentiation of human ventral midbrain dopaminergic neurons from human pluripotent stem cells. Human pluripotent stem cells (hPSCs) have been used for the differentiation of ventral midbrain dopaminergic (vmDA) progenitors and neurons. **(A)** Schematic diagram of monolayer (2D)-based differentiation protocols (Brederlau et al., 2006; Sonntag et al., 2007; Hargus et al., 2010; Kriks et al., 2011; Rhee et al., 2011; Gonzalez et al., 2015; Han et al., 2015; Steinbeck et al., 2015; Gonzalez et al., 2016; Niclis et al., 2017; Wakeman et al., 2017; Adler et al., 2019; Chang et al., 2019; Leitner et al., 2019; Zygogianni et al., 2019; Gantner et al., 2020; Hoban et al., 2020; Mousavinejad et al., 2020; Schweitzer et al., 2020; Song et al., 2020; Kim et al., 2021b; de Luzy et al., 2021; Piao et al., 2021). **(B)** Schematic diagram of embryonic body (EB)-based differentiation protocols (Schulz et al., 2004; Cho et al., 2008; Geeta et al., 2008; Cai et al., 2010; Swistowski et al., 2010; Sanchez-Danes et al., 2012; Grealish et al., 2014; Wakeman et al., 2014; Effenberg et al., 2015; Grealish et al., 2015; Samata et al., 2015; Adil et al., 2017; Xia et al., 2017; Fathi et al., 2018; Tiklova et al., 2020). **(C)** Schematic diagram of neurosphere-based differentiation protocols (Ben-Hur et al., 2004; Cho et al., 2011; Kikuchi et al., 2011; Doi et al., 2012; Ogura et al., 2013; Doi et al., 2014; Komatsu et al., 2015; Katsukawa et al., 2016; Nishimura et al., 2016; Samata et al., 2016; Kikuchi et al., 2017a; Kikuchi et al., 2017b; Francis et al., 2020; Goggi et al., 2020; Miyawaki et al., 2020; Xiong et al., 2021).

**FIGURE 2:**
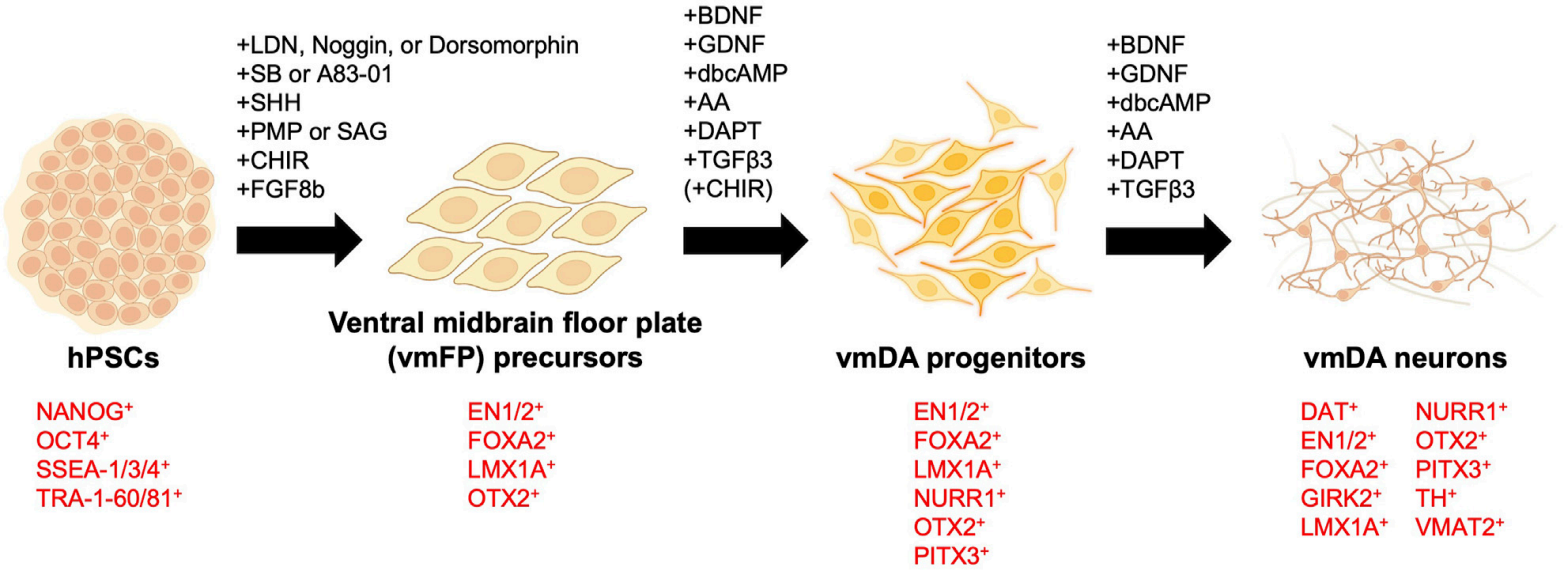
Key small molecules and markers in ventral midbrain dopaminergic neuron differentiation. Human pluripotent stem cells (hPSCs) are differentiated into ventral midbrain lineage cells using specific small molecules. The diagram indicates the small molecules and key molecular markers involved at each developmental stage. AA, ascorbic acid. CHIR, CHIR99021. dbcAMP, dibutyryl-cyclic AMP. LDN, LDN193189. PMP, purmorphamine. SAG, smoothened agonist. SB, SB431542. vmDA, ventral midbrain dopaminergic.

When differentiating hPSCs into vmDA neurons, one of the first considerations is choosing between adherent culture or suspension culture methods. Historically, vmDA progenitors were primarily differentiated either by co-culturing with feeder cells such as MS5, S2, and PA6 stromal cell lines in adherent culture (Ben-Hur et al., 2004; Perrier et al., 2004; Brederlau et al., 2006; Sonntag et al., 2007), or through the formation of embryoid bodies (EBs) in suspension culture (Cho et al., 2008; Geeta et al., 2008; Cai et al., 2010). Adherent culture involves co-culturing hPSCs with feeder cells or using a specially-coated dish to support the adhesion and subsequent differentiation of hPSCs into vmDA neurons in a two-dimensional (2D) cell culture format (Figure 1A). In contrast, the suspension culture method employs the culturing of hPSCs in a suspended state, leading to the differentiation into vmDA neurons *via* the formation of EBs (Figure 1B). Recently, a three-dimensional (3D) method involving the formation of neurospheres for vmDA neuron differentiation has also been introduced (Figure 1C). For ease of description, these culture methods can conveniently be categorized as 2D-based, EB-based, or neurosphere-based differentiation protocols (Figure 1). Currently, the aforementioned methods are extensively employed in various protocols for differentiating vmDA neurons (Table 1). Numerous protocols have been continuously developed over the years to differentiate cells into vmDA neurons by manipulating specific signaling pathways. These protocols use various cell culture techniques and adjust the duration and concentration of a range of small molecules (Figure 2). One notable protocol is the dual-SMAD inhibition developed by the Studer group, which uses Noggin and SB431542 to inhibit BMP and TGF-beta signaling, respectively (Chambers et al., 2009). Additionally, protocols employing small molecules that modulate cell signaling pathways, such as WNT, SHH, and FGF, are also being developed (Castelo-Branco et al., 2004; Joksimovic et al., 2009; Xi et al., 2012). A method combining dual-SMAD inhibition with other small molecules aims to activate these major signaling pathways at varying concentrations and durations. CHIR99021 (CHIR) is used to activate canonical WNT signaling (Lyashenko et al., 2011), while recombinant SHH protein and SHH agonists like purmorphamine are used for SHH signaling. FGF signaling is triggered using the recombinant protein FGF8b (Figure 2). This combination results in a high yield of vmDA progenitor cells capable of differentiating into vmDA neurons (Cai et al., 2010; Kriks et al., 2011; Doi et al., 2012; Sanchez-Danes et al., 2012; Doi et al., 2014; Grealish et al., 2014; Grealish et al., 2015; Samata et al., 2015; Steinbeck et al., 2015; Samata et al., 2016; Adil et al., 2017; Niclis et al., 2017; Nolbrant et al., 2017; Wakeman et al., 2017; Xia et al., 2017; Fathi et al., 2018; Adler et al., 2019; Chang et al., 2019; Zygogianni et al., 2019; Mousavinejad et al., 2020; Schweitzer et al., 2020; Song et al., 2020; Kim T. W. et al., 2021; Xiong et al., 2021). Researchers like Perrier and his colleagues have focused on differentiation using SHH and FGF8 and found that approximately 60%–80% of beta tubulin III (TUJ1)-positive neurons express TH (Perrier et al., 2004). The Studer and Parmar teams have also successfully differentiated vmDA progenitors by modulating similar signaling pathways (Kriks et al., 2011; Kirkeby et al., 2012). More recently, advanced protocols have been introduced to enhance differentiation efficacy through specific 'boosting methods'. The first method involves using 0.7 μM CHIR from day 0, elevating it to 7.5 μM from day 4, and then reducing it to 3 μM from day 7 (Kim T. W. et al., 2021). The second method administers 0.8 μM CHIR from day 2 and doubles the concentration to 1.6 μM from day 11 (Kim S. W. et al., 2021). Due to variations in cell culture methods, small molecule combinations, and timing, different groups have reported varying rates of vmDA neuronal differentiation (Table 1).

### 3.4 Quality over quantity: Why lineage and quality checks are crucial in vmDA cell transplantation

Transplanting vmDA progenitors without prior lineage confirmation can lead to complications, reminiscent of early challenges in transplanting fetal ventral mesencephalon cells without proper verification. Thus, it's crucial to validate the lineage of hPSC-derived vmDA progenitors before their transplantation. Numerous markers, such as FOXA2, LMX1A, OTX2, CORIN, EN1, NURR1, PITX3, TH, VMAT2 as well as GIRK2 and DAT—which are co-expressed in DA neurons in the A9 region—are employed for lineage confirmation (Figure 2). A number of research groups employ the co-expression of FOXA2 and LMX1A as indicators of vmDA progenitors (Table 1). Alongside these two established vmDA progenitor markers, numerous others are in use, including OTX2, CORIN, a precursor marker for vmFP, and EN1 (Kriks et al., 2011; Xi et al., 2012) (Table 1). To confirm that vmDA progenitor cells have differentiated into functional vmDA neurons of the SNpc suitable for cell therapy, a neuron maturation process was carried out for validation. As part of this validation, the following markers were employed: NURR1, which is essential for the generation and maintenance of vmDA neurons; PITX3, which plays a pivotal role in the survival of vmDA neurons; the vmDA neuron-specific marker TH; and the pan-neuronal marker TUJ1 (Table 1). Additionally, several markers, such as GIRK2 and DAT, which are co-expressed in DA neurons within the A9 region, were utilized to confirm the vmDA neuronal lineage in this area (Table 1). The identified neurons were further confirmed to possess the same electrophysiological properties as the vmDA neurons in the A9 region (Schulz et al., 2004; Brederlau et al., 2006; Cho et al., 2008; Cho et al., 2011; Kriks et al., 2011; Rhee et al., 2011; Sanchez-Danes et al., 2012; Doi et al., 2014; Steinbeck et al., 2015; Samata et al., 2016; Kikuchi et al., 2017a; Kikuchi et al., 2017b; Niclis et al., 2017; Wakeman et al., 2017; Leitner et al., 2019; Zygogianni et al., 2019; Song et al., 2020). This collective evidence establishes that the progenitor cells have been successfully directed to differentiate into cells exhibiting characteristics consistent with the vmDA neuronal lineage.

However, when generating mature vmDA progenitor cells through various differentiation protocols, a small fraction may diverge into other neuronal types such as noradrenergic, adrenergic, GABAergic, glutamatergic, and serotonergic neurons, or even into glial cells (Ben-Hur et al., 2004; Cho et al., 2008; Geeta et al., 2008; Cai et al., 2010; Sanchez-Danes et al., 2012; Samata et al., 2016; Adil et al., 2017; Wakeman et al., 2017; Fathi et al., 2018; Chang et al., 2019; Zygogianni et al., 2019). Contaminants like undifferentiated stem cells and proliferating NPCs can also be present, posing a risk of neoplastic mass formation upon transplantation (Roy et al., 2006). Most research groups adopt their own QC measures, often focusing on confirming successful differentiation and functional potential post-transplantation. Animal-based transplantation experiments also serve as a form of QC prior to human clinical trials (Table 2, Table 3). Currently, there is a gap in the availability of standardized or rapid methods for 'pass/fail' assessments to eliminate inappropriate cells immediately prior to transplantation. This 'Last-Mile QC' represents a crucial safety step that would significantly improve the reliability of cell transplantation procedures. While methods like CORIN-positive cell sorting have shown promise—indeed, a study has demonstrated that CORIN-positive cells, once purified, were successfully transplanted, engrafted without tumor formation, and led to behavioral recovery in PD animal models (Doi et al., 2014)—they fall short of meeting the full requirements of 'Last-Mile QC.' One significant limitation is that CORIN-based sorting generally necessitates an additional 16 days of *in vitro* culture post-sorting, leaving an unresolved issue in the existing QC process (Doi et al., 2014; Katsukawa et al., 2016; Nishimura et al., 2016; Samata et al., 2016; Kikuchi et al., 2017a; Kikuchi et al., 2017b). Therefore, rigorous QC measures should be in place at the vmDA progenitor cell stage. To ensure more predictable and effective PD cell therapy outcomes, it is imperative to either develop a new protocol for lineage verification using specific markers or invent new technologies capable of real-time tracking of cell differentiation. By implementing such stringent QC, we can effectively isolate the correct vmDA progenitor cells for transplantation, thereby mitigating risks associated with cell therapy.

**TABLE 2:** Summary of graft verification following human pluripotent stem cell-derived dopaminergic neuronal transplantation.

Injected cell type (DIV)	Injected site (cell number)	Graft verification	Note	ID*	Ref
DA neuron (22)	Mouse_Str (0.012 M)	TH (IHC)	After 13 weeks, the SAPNS-encapsulated-cell-transplanted group demonstrated a 32% increase in TH^+^ cells compared to the untreated-cell-transplanted group	a1	Francis et al. (2020)
DA progenitor (22)	Mouse_Str (0.2 M)	TH, TUJ1 (IHC)	After 8 weeks, the DAPT- or compound E-treated-cell-transplanted group exhibited an increase of 0.9 ± 0.5% or 1.6 ± 0.8% TH^+^/HNA^+^ cells, respectively, compared to the untreated-cell-transplanted group	b40	Ogura et al. (2013)
vmDA neuron (25)	Mouse_Str (0.2 M)	TH (IHC)	TH^+^ cells detected after 4 months	a2	Leitner et al. (2019)
vmDA neuron (25)	Mouse_Str (0.2 M)	FOXA2, TH (IHC); Pacemaking activity (WCPC)	TH^+^ cells detected after 4-6 months	a3	Steinbeck et al. (2015)
vmDA progenitor (16)	Mouse_SN (0.075 M) or Str (0.15 M)	FOXA2, TH (IHC)	FOXA2^+^TH^+^ cells detected after 6 months	b41	Adler et al. (2019)
vmDA progenitor (32)	Mouse_SN or Str (0.05 M)	FOXA2, GIRK2, LMX1A, TH (IHC); Action potentials (WCPC)	GIRK2^+^TH^+^, FOXA2^+^, LMX1A^+^ cells identified, along with grafted cell fibers extending to the caudate putamen, amygdala, substantia innominata, and cortex detected, after 4 months	a4	Xiong et al. (2021)
vmDA progenitor (25)	Mouse_Str (0.15 M)	FOXA2, TH (IHC)	After 4.5 months, detected TH^+^FOXA2^+^ cells and less than 1% of Ki67^+^ cells	a5	Kriks et al. (2011)
vmDA progenitor (25)	Mouse_Str (0.05 M)	FOXA2, GFP [ = LMX1A], OTX2 (IHC)	High OTX2 and FOXA2 expression detected at 1-month post-implantation in both xenogeneic and xeno-free grafts, with a non-significant trend favoring xeno-free composition; significant phenotypic differences only revealed *via* LMX1A-eGFP reporter, showing 2.5-fold more GFP [LMX1A]^+^ cells in xeno-free than in xenogeneic grafts	b21	Niclis et al. (2017)
vmDA progenitor (16)	Mouse_Str (0.2 M)	ALDH1A1, GIRK2, TH (IHC)	GIRK2^+^TH^+^ and ALDH1A1^+^TH^+^GIRK2^+^ cells detected, along with ALDH1A1^+^ cell fibers extending to the Str, after 6 months	b24	Kim et al. (2021b)
vmDA progenitor (21-GFP [ = LMX1A]^+^ sorted at DIV21)	Mouse_Str (0.1 M)	DA (HPLC); CCK, GFP [ = LMX1A], TH (IHC); *AADC, ALDH1A2, CCK, DLK1, TH, VMAT2* (qRT-PCR); *ADCYAP1, ALDH1A2, CALB1, CCK, DDC, DLK1, OSBP2, RET, TH, VMAT2* (RNA-seq)	GFP [ = LMX1A]^+^TH^+^ and CCK^+^TH^+^ cells detected after 6 months	b29	Gantner et al. (2020)
vmDA progenitor (28)	Mouse_Str (0.1 M)	TH (IHC)	TH^+^ cells detected after 2 weeks	b43	Schweitzer et al. (2020)
vmDA progenitor (35-LMX1A OE)	Mouse_Str (0.2 M)	DAT, GIRK2, TH, TUJ1 (IHC)	TH^+^TUJ1^+^, TH^+^DAT^+^, and TH^+^GIRK2^+^ cells detected, with an absence of GABAergic or serotonergic neurons observed, after 5 months	b42	Sanchez-Danes et al. (2012)
vmDA progenitor (30-NCAM^+^ sorted at DIV28)	Mouse_Str (0.2 M)	TH, TUJ1 (IHC)	After 12 weeks of transplantation with either healthy or SNCA^p.A53T/+^ PD hiPSC-derived cells, TH^+^ cells detected	b44	Zygogianni et al. (2019)
vmDA progenitor (28-CORIN^+^ sorted at DIV12)	Mouse_Str (0.2 M)	n/a	After 6 months, no pathological alpha-synuclein accumulation observed in either healthy or PD hiPSC-derived vmDA progenitor-transplanted group	b34	Kikuchi et al. (2017b)
NSC (32-34)	Rat_Str (n/a)	TH (IHC)	After 12 weeks, TH^+^ cells were detected with no evidence of teratoma formation	a6	Swistowski et al. (2010)
NSC (19)	Rat_Str (0.5-0.6 M)	TH (IHC)	After 12 weeks, the group transplanted with lenti-MEF2CA-infected NSCs showed a higher proportion of TH^+^ cells in the Str compared to the control-infected NSC group (15.1 ± 1.7% vs. 1.9 ± 0.9%)	a7	Cho et al. (2011)
NSC (14-18)	Rat_Str (0.5 M)	TH, TUJ1 (IHC)	TH^+^TUJ1^+^ cells detected after 16 weeks	a8	Han et al. (2015)
NSC (46)	Rat_Str (0.5 M)	DA (ELISA); GIRK2, TH, VMAT2 (IHC)	TH^+^VMAT2^+^ and GIRK2^+^TH^+^ cells detected after 28 weeks	b45	Gonzalez et al. (2015)
DA neuron (33)	Rat_Str (0.4 M)	DAT, TH, TUJ1 (IHC)	After 12 weeks, detected TH^+^ and DAT^+^ cells with no evidence of teratoma formation	a9	Cho et al. (2008)
DA neuron (33)	Rat_Str (0.1 M)	TH (IHC)	After 3 weeks, detected TH^+^ cells in both protocol 1 and protocol 2 conditions	b46	Effenberg et al. (2015)
DA neuron (42-NCAM^+^ sorted at DIV42 or unsorted)	Rat_Str (0.2-0.4 M)	TH, GIRK2 (IHC)	After 16 weeks of transplantation with PD hiPSC-derived cells, detected TH^+^GIRK2^+^ cells with no tumors observed	a10	Hargus et al. (2010)
DA progenitor (40-45)	Rat_SN (1.2 M)	GIRK2, MAP2, TH, TUJ1 (IHC)	TH^+^MAP2^+^ and TH^+^GIRK2^+^ cells detected after 48 weeks	a11	Geeta et al. (2008)
DA progenitor (42)	Rat_Str (0.1 M)	GIRK2, TH (IHC)	GIRK2^+^TH^+^ (data not shown) and TH^+^ cells detected after 3 months	a12	Sonntag et al. (2007)
DA progenitor (16, 20, 23)	Rat_Str (0.1 M)	TH (IHC)	At 2 weeks, DIV23-cell-transplants showed lower survival rates than DIV16-cell-transplants (*p* < 0.05), TH^+^ cells detected, and no NG2^+^, GAD^+^, or CHAT^+^ cells found; teratoma formation occurred with DIV16 and DIV20 cells but not with DIV23 cells	a13	Brederlau et al. (2006)
DA progenitor (56)	Rat_Str (0.4 M)	DAT, TH (IHC); *EN1, TH, AADC* (RT-PCR)	DAT^+^ and TH^+^ cells detected after 12 weeks	a14	Ben-Hur et al. (2004)
DA progenitor (19)	Rat_Str (0.44 M)	FOXA2, TH (IHC)	After 14 weeks of transplantation, detected TH^+^ cells and observed a significant reduction in Ki67^+^/HNA^+^ cells due to gamma-ray irradiation (*p* < 0.01)	b47	Katsukawa et al. (2016)
DA progenitor (30)	Rat_Str (0.001-0.02 M)	MAP2, TH (IHC)	MAP2^+^ (data not shown) and TH^+^ cells detected after 8 weeks	b48	Schulz et al. (2004)
DA progenitor (41)	Rat_Str (0.1 M)	TH, TUJ1 (IHC)	After 1 week of transplantation, detected TH^+^ and TUJ1^+^ cells with neurites extending from the graft into the host brain	b49	Komatsu et al. (2015)
vmDA neuron (33, 38)	Rat_Str (0.45 M)	FOXA2, GIRK2, TH (IHC)	After 6 months, TH^+^FOXA2^+^ and GIRK2^+^TH^+^ cells detected, transplanted cell fibers extending to the parenchyma, and a few CHAT^+^ and 5-HT^+^ cells identified	a15	Wakeman et al. (2017)
vmDA neuron (25)	Rat_Str (0.1 M)	FOXA2, TH, TUJ1 (IHC)	After 4.5 months of transplantation, detected TH^+^FOXA2^+^ cells with higher percentages of TH^+^ cells in the 3D HA-hep-RGD hydrogel-encapsulated group compared to the 2D cell suspension group (∼7% vs. 1.3% of total transplanted cells; ∼85% vs. ∼80% of surviving cells)	b50	Adil et al. (2017)
vmDA progenitor (16)	Rat - Midbrain (0.075 M)	TH (IHC)	After 24 weeks, TH^+^ cells and transplanted cell fibers were detected extending to various brain regions, including A9 target (Str) and A10 targets (prefrontal cortex, nucleus accumbens, septum, ventral Str), as well as the thalamus and hypothalamus	a16	Cardoso et al. (2018)
vmDA progenitor (16)	Rat_SN or Str (0.1 M)	DAT, GIRK2, NCAM, TH (IHC); [^18^F]fallypride, [^18^F]LBT999 (PET)	[hESC-derived cell transplanted to Str] After 6 months, DAT^+^ and TH^+^GIRK2^+^ cells detected with transplanted cell fibers extending into the Str	a17	Grealish et al. (2014)
			[hESC-derived cell transplanted to SN] After 6 months, TH^+^GIRK2^+^ cells detected with transplanted cell fibers extending into multiple regions including the amygdala, dorsolateral striatum, piriform cortex, ventral striatum, olfactory tubercle, lateral septum, and frontal lobe		
			[OTX2 OE hESC-derived cell transplanted to SN] After 6 months, OTX2 OE hESC-derived NCAM^+^ cells showed less innervation in the A9 target structure compared to both parental hESC-derived and fetal-derived cells		
vmDA progenitor (22-36)	Rat_Str (1 M)	ALDH1A1, LMX1A, TH (IHC)	After 6 weeks, detected ALDH1A1^+^TH^+^, LMX1A^+^ALDH1A1^+^ cells, Ki67^+^ cells, and a teratoma-like structure	a18	Cai et al. (2010)
vmDA progenitor (35-63, 40-68)	Rat_Str (0.3-0.75 M)	EN1, NURR1, TH, TUJ1, VMAT2 (IHC)	[0.75 M cells injected] After 8 weeks, TH^+^ and Ki67^+^ cells as well as rosette structures detected; tumors observed in grafted animals within the same timeframe	a19	Rhee et al. (2011)
			[0.3 M cells injected] After 8 weeks of transplantation, TH^+^, TH^+^VMAT2^+^, TH^+^NURR1^+^, and TH^+^EN1^+^ cells detected without rosette formation		
			[0.3 M cells (5 days more matured) injected] No detected TH^+^ cells		
vmDA progenitor (16)	Rat_Str (0.3 M)	TH (IHC)	After 24 weeks, detected TH^+^ cells with transplanted cells exhibiting synaptic connections with neighboring neurons	a20	Grealish et al. (2015)
vmDA progenitor (25)	Rat_Str (0.05 M)	FOXA2, GFP [ = PITX3], TH (IHC)	After 1 month of transplantation, FOXA2^+^TH^+^GFP [ = PITX3]^+^ cells detected; after 6 months, TH^+^GFP [ = PITX3]^+^ cells detected	a21	Niclis et al. (2017)
vmDA progenitor (24)	Rat_Str (0.45 M)	DAT, FOXA2, LMX1A, NURR1, TH (IHC)	After 8 weeks of transplantation with LIN28A^corrected/+^ PD hiPSC-derived cells, TH^+^LMX1A^+^, TH^+^FOXA2^+^, TH^+^DAT^+^, and TH^+^NURR1^+^ cells were detected	a22	Chang et al. (2019)
vmDA progenitor (25)	Rat_Str (0.4 M)	EN1, FOXA2, GIRK2, LMX1A, TH (IHC); [^18^F]FBCTT, [^18^F]fallypride (PET)	After 6 months of transplantation, EN1^+^TH^+^, TH^+^LMX1A^+^, TH^+^FOXA2^+^, and GIRK2^+^TH^+^ cells were detected, and DA release from transplanted cells was confirmed	a23	Goggi et al. (2020)
vmDA progenitor (16)	Rat_Str (0.45 M)	TH (IHC)	TH^+^ cell fibers extending to the Str observed after 5.5 months of transplantation	a24	Kim et al. (2021b)
vmDA progenitor (16)	Rat_Str (0.4 M)	FOXA2, TH (IHC)	TH^+^ and FOXA2^+^ cells detected after 8 months	a25	Piao et al. (2021)
vmDA progenitor (19, 25)	Rat_Str (0.125 M)	GIRK2, TH (IHC)	After 26 weeks, detected GIRK2^+^TH^+^ cells in DIV19 transplants, but DIV25 donor cells showed significantly fewer TH^+^ cells and reduced capacity to innervate the host Str (*p* < 0.001)	a26	de Luzy et al. (2021)
vmDA progenitor (28)	Rat_Str (0.1M)	ALDH1A1, CALB, DAT, FOXA2, GIRK2, LMX1A, NCAM, NURR1, TH (IHC)	After 26 weeks post-transplantation of PD hiPSC-derived cells, observed were 70%–80% FOXA2^+^LMX1A^+^/TH^+^, >90% NURR1^+^/TH^+^, and DAT^+^TH^+^ and GIRK2^+^TH^+^ cells; no rosettes or teratomas; TH^+^ neurons predominantly co-expressed GIRK2 over CALB, with additional A9 markers like ALDH1A1, often alongside SOX6 and GIRK2 for A9-type vmDA neurons, while some co-expressed CALB for A10-type vmDA neurons	a27	Song et al. (2020)
vmDA progenitor (16)	Rat_Str (0.3 M) or SN (0.15 M)	TH (IHC); *DDC, DLK1, FOXA2, LMX1A, GFRA1, NURR1, OTX2, PBX1, PITX3, RET, TH, VMAT2* (scRNA-seq)	[Transplanted to Str] After 6 months, detected TH^+^ cells with engrafted cells extending to the dorsolateral Str and prefrontal cortex	a28	Tiklova et al. (2020)
			[Transplanted to SN] After 9 months, detected TH^+^ cells		
vmDA progenitor (25)	Rat_Str (0.25 M)	DAT, FOXA2, GIRK2, NURR1, PITX3, TH (IHC)	After 5 months, detected TH^+^FOXA2^+^, TH^+^PITX3^+^, TH^+^NURR1^+^, DAT^+^TH^+^, and GIRK2^+^TH^+^ cells, with transplanted cell fibers extending to the Str	a5	Kriks et al. (2011)
vmDA progenitor (25)	Rat_Str (0.3 M)	NURR1, TH (IHC)	NURR1^+^ and TH^+^ cells detected after 3 months	b51	Xia et al. (2017)
vmDA progenitor (16)	Rat_Str (0.3 M)	GIRK2, PITX3, TH (IHC)	After 12-18 weeks, detected GIRK2^+^, PITX3^+^, TH^+^ cells, with engrafted cells extending to various brain regions	b52	Hoban et al. (2020)
vmDA progenitor (16)	Rat_Str (0.15 M)	TH (IHC)	After 91 or 127 days of transplantation, TH^+^ cells detected	b53	Mousavinejad et al. (2020)
vmDA progenitor (21-GFP [ = LMX1A]^+^ sorted at DIV21)	Rat_Str (0.1 M)	CALB, FOXA2, GFP [ = LMX1A], GIRK2, TH (IHC)	TH^+^FOXA2^+^, TH^+^CALB^+^GIRK2^+^, and TH^+^GFP [ = LMX1A]^+^ cells detected, along with TH^+^ cell fibers extending to the Str, after 26 weeks	a29	Gantner et al. (2020)
vmDA progenitor (21-GFP [ = PITX3]^+^ sorted at DIV21)	Rat_Str (0.1 M)	CALB, GIRK2, GFP [ = PITX3], TH (IHC)	CALB^+^GIRK2^+^GFP [ = PITX3]^+^, TH^+^GFP [ = PITX3]^+^ cells detected after 6 months		
vmDA progenitor (12-CNTN2^+^ sorted at 12 DIV)	Rat_Str (0.14-0.16 M)	DAT, TH, TUJ1 (IHC)	After 12 weeks, detected TH^+^TUJ1^+^ and DAT^+^TH^+^ cells, observed no tumor formation, and noted a significant increase in %TH^+^ and %DAT^+^ in CNTN2^+^ sorted cell transplants compared to unsorted cell transplants (*p* < 0.01)	a30	Fathi et al. (2018)
vmDA progenitor (28-LRTM1^+^ sorted at DIV14)	Rat_Str (0.13 M)	FOXA2, GIRK2, NURR1, TH (IHC)	After 12 weeks of transplantation, detected GIRK2^+^TH^+^, FOXA2^+^TH^+^, and NURR1^+^TH^+^ cells; TH^+^ cells were four times more abundant in LRTM1^+^ sorted cells compared to unsorted cells, with significantly higher percentages of TH^+^HNA^+^, FOXA2^+^HNA^+^, and NURR1^+^HNA^+^ cells in the LRTM1^+^ sorted group (*p* < 0.001, *p* < 0.001, *p* < 0.01, respectively); transplanted cell fibers extended into the host brain	a31	Samata et al. (2016)
vmDA progenitor (28-CORIN^+^ sorted at DIV12)	Rat_Str (0.4 M)	FOXA2, GIRK2, NURR1, PITX3, TH (IHC)	After 16 weeks of transplantation, detected FOXA2^+^TH^+^, PITX3^+^TH^+^, NURR1^+^TH^+^, and GRIK2^+^TH^+^ cells; TH^+^ cells were about twice as abundant in CORIN^+^ sorted cells compared to unsorted cells, with a significantly higher percentage of TH^+^/HNA^+^ cells in the CORIN^+^ sorted group (*p* < 0.001)	a32	Doi et al. (2014)
vmDA progenitor (28-CORIN^+^ sorted at DIV12)	Rat_Str (0.4 M)	FOXA2, TH (IHC)	After 16 weeks of transplantation, TH^+^FOXA2^+^ cells were detected and TH^+^ neuronal fibers extended to DARPP32^+^ striatal neurons	a33	Nishimura et al. (2016)
vmDA progenitor (28-CORIN^+^ sorted at DIV12)	Rat_Str (0.4 M)	FOXA2, TH (IHC)	After 4 months of transplantation, FOXA2^+^TH^+^ cells and cell fibers extending to the Str detected in both healthy and PD hiPSCs-derived groups	a34	Kikuchi et al. (2017b)
vmDA progenitor (28-CORIN^+^ sorted at DIV12)	Rat_Str (0.4 M)	NURR1, TH (IHC)	After 16 weeks, NURR1^+^TH^+^ cells detected and TH^+^ cell fibers extended to the Str	a35	Samata et al. (2015)
vmDA progenitor (28-CORIN^+^ sorted at DIV12)	Rat_Str (0.5 M)	FOXA2, NURR1, TH (IHC)	After 1 month, NURR1^+^TH^+^ and FOXA2^+^ cells were detected, with a higher percentage of NURR1^+^TH^+^/HNA^+^ cells in the 60 mg/kg Zonisamide-treated group compared to the vehicle group (*p* < 0.01); after 4 months, NURR1^+^TH^+^ and FOXA2^+^ cells were detected, with no significant difference in the percentage of TH^+^NURR1^+^/HNA^+^ cells between the 60 mg/kg Zonisamide-treated and vehicle groups	b54	Miyawaki et al. (2020)
NSC (46)	Monkey_SN & Str (10 or 20 M)	DA (HPLC); TH (IHC)	After 12 months, the low-dose group had the highest combined DA concentrations across all brain regions (n.s.) and superior DA neuron innervation in the Str, while both low-dose and high-dose groups exhibited more TH^+^ cells in the SN than the control (47,507 ± 5,555 and 49,028 ± 4,039 vs 20,549 ± 1,252 cells)	a36	Gonzalez et al. (2016)
NSC (46)	Monkey_SN & Str (8 M)	DA (ELISA); GIRK2, TH, VMAT2 (IHC)	After 14 weeks, TH^+^, GIRK2^+^, and VMAT2^+^ cells detected, no tumor formation observed, and higher DA levels in NSC transplantation condition compared to vehicle control	b45	Gonzalez et al. (2015)
DA neuron (57)	Monkey_SN & Str (4.5 or 6.25 M)	TH, TUJ1 (IHC)	After 6 weeks, no TH^+^ cells detected, but transplanted cell fibers innervated the corpus callosum, putamen, griseum pontis caudato lenticulares, and lateral ventricle	b55	Wakeman et al. (2014)
DA progenitor (28 [R}/42 [L])	Monkey_Str (4.8 M)	DAT, GIRK2, NURR1, PITX3, TH, VMAT2 (IHC); [^18^F]DOPA, [^11^C]DTBZ, [^11^C]PE2I (PET)	After 6 months, NURR1^+^TH^+^, VMAT2^+^TH^+^, DAT^+^TH^+^, GIRK2^+^TH^+^, and PTIX3^+^TH^+^ cells detected, with TH^+^ cells predominantly distributed at graft periphery; more total TH^+^ cells in left Str compared to right (right 30.7 K vs. left 126 K)	a37	Kikuchi et al. (2011)
vmDA neuron (33, 38)	Monkey_Str (3.75 M)	FOXA2, GIRK2, TH (IHC)	TH^+^FOXA2^+^ and TH^+^GIRK2^+^ cells detected after 3 months	b15	Wakeman et al. (2017)
vmDA progenitor (25)	Monkey_Str (7.5 M)	TH, FOXA2 (IHC)	FOXA2^+^TH^+^ cells detected after 1 month	a5	Kriks et al. (2011)
vmDA progenitor (42)	Monkey_Str (4.8 M)	AADC, PITX3, TH, TUJ1, VMAT2 (IHC); [^18^F]DOPA (PET)	After 12 months, grafted cells innervated the host putamen and exhibited TH^+^VMAT2^+^, TH^+^AADC^+^, and TH^+^PITX3^+^ cells	a38	Doi et al. (2012)
vmDA progenitor (15 [R]/21 [L] or 28 [R]/35 [L]-LRTM1^+^ sorted at DIV14)	Monkey_Str (4 M)	DAT, FOXA2, GIRK2, NURR1, PITX3, TH (IHC)	After 12 weeks, grafts derived from DIV28 cells showed the largest number of TH^+^ cells, which extended TH^+^ neuronal fibers into the host brain, predominantly co-expressed FOXA2, NURR1, and PITX3, and some were large in size and expressed DAT and GIRK2	b31	Samata et al. (2016)
vmDA progenitor (28-CORIN^+^ sorted at DIV12)	Monkey_Str (4.8 M)	DAT, FOXA2, GIRK2, TH (IHC); [^18^F]DOPA, [^11^C]PE2I (PET)	After 8-24 months, surviving grafted dopaminergic neurons exhibited neurite extension throughout the putamen and partially into the caudate head, with morphology and size resembling host substantia nigra neurons; most cells expressed FOXA2, 33.3 ± 24.4% co-expressed TH, and the average number of TH^+^ neurons was 64 ± 49 K per hemisphere, with no difference between healthy and PD-derived grafts; these TH^+^ cells also expressed DAT and GIRK2, and no 5-HT^+^ cells were observed due to CORIN-based cell sorting	a39	Kikuchi et al. (2017a)

* Papers with IDs a1 to a39 assessed the impact of cell transplantation on motor function recovery, while papers with IDs b40 to b55 did not assess the effects of motor function recovery after cell transplantation. Please note that papers b15, b21, b24, b29, b31, and b34 correspond, respectively, to papers a15, a21, a24, a29, a31, and a34.

** DA, dopamine; DIV, days *in vitro*; ELISA, confirmed by enzyme-linked immunosorbent assay; HPLC, confirmed by high performance liquid chromatography; IHC, confirmed by immunohistochemistry. L, left. n/a, not available. n. s., not significant. NSC, neural stem cell; OE, overexpression; PD, Parkinson’s disease. PET, confirmed by positron emission tomography scan. R, right. RNA-seq, confirmed by RNA, sequencing; RT-PCR, confirmed by reverse transcription PCR. C62:H68scRNA-seq, confirmed by single cell RNA-seq. SN, substantia nigra. Str, striatum. vmDA, ventral midbrain dopaminergic; WCPC, confirmed by whole-cell patch-clamp recording.

**TABLE 3:** Summary of behavioral recovery after human pluripotent stem cell-derived dopaminergic neuronal transplantation.

PD model	Injected cell type_site	Behavioral test method	Result	ID	Ref
Mouse (6-OHDA)	DA neuron_Str	ApIR, cylinder, rotarod	After 12 weeks, the SAPNS-encapsulated cell-transplanted group exhibited significant reductions in rotations during the ApIR test (*p* < 0.05), prolonged stay time in the rotarod test (*p* < 0.001), and an increased contralateral forelimb use in the cylinder test (n.s.)	a1	Francis et al. (2020)
Mouse (6-OHDA)	vmDA neuron_Str	AmIR	Decreased rotations in the AmIR test after 8 weeks (no statistical analysis data available)	a2	Leitner et al. (2019)
Mouse (6-OHDA)	vmDA neuron_Str	AmIR, corridor	After 16 weeks, decreased rotations in the AmIR test (no statistical analysis data available), accompanied by a significant improvement in the corridor test (vs. pre-transplantation, *p* < 0.01)	a3	Steinbeck et al. (2015)
Mouse (6-OHDA)	vmDA progenitor_SN or Str	AmIR, cylinder, rotarod	After 6 months of either nigral or striatal grafting, significant reductions in rotations during the AmIR test (*p* < 0.001), accompanied by noteworthy enhancements in both the cylinder and rotarod tests (*p* < 0.001, each)	a4	Xiong et al. (2021)
Mouse (6-OHDA)	vmDA progenitor_Str	AmIR	After 16 weeks, significant reductions in rotations during the AmIR test (vs. rosette-derived grafts, *p* < 0.01)	a5	Kriks et al. (2011)
Rat (6-OHDA)	NSC_Str	AmIR	After 12 weeks, significant reductions in rotations during the AmIR test (*p* < 0.05)	a6	Swistowski et al. (2010)
Rat (6-OHDA)	NSC_Str	ApIR, cylinder	Transplanting lenti-MEF2CA-infected NSCs led to significant reductions in rotations during the AmIR test after 8 weeks (vs. transplanting control-infected NSCs, *p* ≤ 0.035), along with a noteworthy improvement in the cylinder test after 9 weeks (vs. transplanting control-infected NSCs, *p* < 0.03)	a7	Cho et al. (2011)
Rat (6-OHDA)	NSC_Str	ApIR, rotarod	After 16 weeks, significant reductions in rotations during the ApIR test (*p* < 0.05) and a notable improvement in the rotarod test (*p* < 0.01)	a8	Han et al. (2015)
Rat (6-OHDA)	DA neuron_Str	AmIR, ApIR, stepping	After 12 weeks, significant reductions in rotations during both the AmIR (58.37 ± 5.9%, *p* < 0.001) and the ApIR (49.43 ± 1.74%, *p* < 0.001) tests, accompanied by a noteworthy enhancement in the stepping adjustments (*p* < 0.001)	a9	Cho et al. (2008)
Rat (6-OHDA)	DA neuron (PD-hiPSC-derived, unsorted)_Str	AmIR, ApIR, cylinder, stepping	After 16 weeks, significant reductions in rotations during both the AmIR and the ApIR tests (*p* < 0.05, each), with no significant changes observed in the cylinder test and the stepping adjustments	a10	Hargus et al. (2010)
	DA neuron (PD-hiPSC-derived, NCAM^+^ sorted)_Str	AmIR, ApIR	After 16 weeks, significant reductions in rotations during the AmIR test (*p* < 0.01), with no significant changes in the ApIR test		
Rat (6-OHDA)	DA progenitor_SN	ApIR, forelimb placing	After 48 weeks, significant reductions in rotations during the ApIR test (*p* < 0.05), along with a significant improvement in the forelimb placing (*p* < 0.05)	a11	Geeta et al. (2008)
Rat (6-OHDA)	DA progenitor_Str	AmIR	After 12 weeks, notable recovery in one rat (WA07-derived cell-transplanted, *n* = 1) with no marked change in others (WA07-derived cell-transplanted, *n* = 8; WA09-derived cell-transplanted, *n* = 5)	a12	Sonntag et al. (2007)
Rat (6-OHDA)	DA progenitor_Str	AmIR	No significant changes in rotation scores after 2, 4, 8, and 13 weeks	a13	Brederlau et al. (2006)
Rat (6-OHDA)	DA progenitor_Str	AmIR, ApIR, forelimb placing, stepping	After 12 weeks, a 31% reduction (ApIR, *p* = 0.0015), and a 45% reduction (AmIR, *p* = 0.001) in rotations, and a significant improvement in both the stepping (*p* = 0.0012) and the forelimb placement (*p* = 0.003) tests	a14	Ben-Hur et al. (2004)
Rat (6-OHDA)	vmDA neuron_Str	AmIR, ApIR	After 6 months, significant reductions in rotations during both the AmIR (*p* < 0.0001) and the ApIR (*p* = 0.0191) tests	a15	Wakeman et al. (2017)
Rat (6-OHDA)	vmDA progenitor_Midbrain	AmIR	After 24 weeks, two out of three rats showed a reduction in rotations during the AmIR test (vs. pre-transplantation, n.s.)	a16	Cardoso et al. (2018)
Rat (6-OHDA)	vmDA progenitor_Str	AmIR	After 16 weeks, significant reductions in rotations during the AmIR test (*p* < 0.01)	a17	Grealish et al. (2014)
Rat (6-OHDA)	vmDA progenitor_Str	AmIR	After 6 weeks, reductions in rotations during the AmIR test (vs. pre-transplantation, data not shown, n.s.)	a18	Cai et al. (2010)
Rat (6-OHDA)	vmDA progenitor_Str	AmIR	[0.75 M cells injected] After 8 weeks, the AmIR decreased to 23.57 ± 5.48% of pre-transplantation scores (*p* < 0.01)	a19	Rhee et al. (2011)
			[0.3 M cells injected] After 8 weeks, the AmIR decreased to 52.46 ± 6.28% of pre-transplantation scores (*p* < 0.01)		
			[0.3 M cells (5 days more matured) injected] After 8 weeks, no significant AmIR reduction observed		
Rat (6-OHDA)	vmDA progenitor_Str	AmIR	After 6 months, significant reductions in rotations during the AmIR test (vs. pre-transplantation, *p* < 0.01)	a20	Grealish et al. (2015)
Rat (6-OHDA)	vmDA progenitor_Str	AmIR	After 6 months, significant reductions in rotations during the AmIR test (*p* < 0.001)	a21	Niclis et al. (2017)
Rat (6-OHDA)	vmDA progenitor_Str	AmIR	Significant decrease in the AmIR observed in LIN28A^corrected/+^ PD hiPSC-derived cell-grafted group compared to LIN28A^p.R192G/+^ PD hiPSC-derived cell-grafted group after 8 weeks (*p* < 0.001)	a22	Chang et al. (2019)
Rat (6-OHDA)	vmDA progenitor_Str	AmIR	Complete behavioral recovery observed in both low-TH and high-TH grafted animals at 6 months post-transplantation, with -14.2 ± 21.2% and -23.9 ± 7.5% reductions in the AmIR relative to pre-transplant levels, respectively (no statistical analysis data available)	a23	Goggi et al. (2020)
Rat (6-OHDA)	vmDA progenitor_Str	AmIR	After 5 months, significant reductions in rotations during the AmIR test (*p* < 0.01)	a24	Kim et al. (2021b)
Rat (6-OHDA)	vmDA progenitor_Str	AmIR	After 8 months, significant reductions in rotations during the AmIR test (*p* < 0.0001)	a25	Piao et al. (2021)
Rat (6-OHDA)	vmDA progenitor_Str	AmIR	After 24 weeks, significant reductions in rotations during the AmIR test (vs. pre-transplantation, *p* < 0.05)	a26	de Luzy et al. (2021)
Rat (6-OHDA)	vmDA progenitor_Str (PD-hiPSC-derived)	AmIR, corridor, cylinder, stepping	[Fresh cell transplanted] After 24 weeks, completely decreased rotations in the AmIR test (*p* < 0.001) and significant improvements in the corridor (*p* < 0.01), cylinder (*p* < 0.001), and stepping (*p* < 0.001) tests	a27	Song et al. (2020)
			[Cryopreserved cell transplanted] After 24 weeks, completely decreased rotations in the AmIR test (*p* < 0.001) and significant improvements in the corridor (*p* < 0.05), cylinder (*p* < 0.001), and stepping (*p* < 0.001) tests		
Rat (6-OHDA)	vmDA progenitor_Str or SN	AmIR, cylinder	[Intrastriatal graft] After 24 weeks, significant reductions in rotations during the AmIR test (vs. pre-transplantation, *p* < 0.001) and significant improvements in the cylinder test (vs. pre-transplantation, *p* < 0.01)	a28	Tiklova et al. (2020)
			[Intranigral graft] After 24 weeks, significant reductions in rotations during the AmIR test (vs. pre-transplantation, *p* < 0.01)		
Rat (6-OHDA)	vmDA progenitor_Str	AmIR, cylinder, stepping	Significant decreases in AmIR test rotations and improvements in stepping adjustments after 18 weeks (*p* < 0.01, each), along with significant cylinder test improvement after 20 weeks (*p* < 0.01)	a5	Kriks et al. (2011)
Rat (6-OHDA)	vmDA progenitor (GFP [ = PITX3]^+^ sorted)_Str	AmIR, cylinder	Significant reductions in AmIR test rotations observed in both uninfected and AAV-GDNF infected (3 weeks before transplantation) rats after 24-week cell transplantation (*p* < 0.001 and *p* < 0.01, respectively), with only the AAV-GDNF infected group showing significant improvement in contralateral paw touches in the cylinder test (*p* < 0.05)	a29	Gantner et al. (2020)
	vmDA progenitor (GFP [ = LMX1A]^+^ sorted)_Str	AmIR, cylinder	Significant reductions in AmIR test rotations observed in both uninfected and AAV-GDNF infected (3 weeks after transplantation) rats after 26-week cell transplantation (*p* < 0.0001 and *p* < 0.001, respectively), with only the AAV-GDNF infected group showing significant improvement in contralateral paw touches in the cylinder test (*p* < 0.0001)		
Rat (6-OHDA)	vmDA progenitor (unsorted)_Str	ApIR, cylinder	After 10 weeks, significant reductions in rotations during the ApIR test (unsorted and CNTN2^+^ sorted cells vs. lesion control, *p* ≤ 0.01), while there were no improvements in cylinder test (n.s.)	a30	Fathi et al. (2018)
	vmDA progenitor (NCAM^+^ sorted)_Str	ApIR, cylinder	Significant reductions in rotations during the ApIR test after 6-12 weeks (*p* < 0.01), along with significant improvements in cylinder test after 8-12 weeks (*p* < 0.01)		
	vmDA progenitor (CNTN2^+^ sorted)_Str	ApIR, cylinder	After 10 weeks, significant reductions in rotations during the ApIR test (vs. unsorted cells, *p* < 0.01; vs. unsorted cells and lesion control, *p* < 0.001), coupled with a substantial improvement in the cylinder test (vs. unsorted cells and lesion control, *p* < 0.0001)		
Rat (6-OHDA)	vmDA progenitor (unsorted)_Str	AmIR, ApIR	After 16 weeks, significant reductions in rotations during both the AmIR and the ApIR (*p* < 0.05, each) tests	a31	Samata et al. (2016)
	vmDA progenitor (LRTM1^+^ sorted)_Str	AmIR, ApIR	After 16 weeks, significant reductions in rotations during both the AmIR (*p* < 0.001) and the ApIR (*p* < 0.05) tests		
Rat (6-OHDA)	vmDA progenitor (unsorted)_Str	AmIR	After 16 weeks, significant reductions in rotations during the AmIR test (*p* = 0.0017)	a32	Doi et al. (2014)
	vmDA progenitor (CORIN^+^ sorted)_Str	AmIR	After 16 weeks, significant reductions in rotations during the AmIR test (*p* = 0.0003)		
Rat (6-OHDA)	vmDA progenitor (CORIN^+^ sorted)_Str	AmIR	After 16 weeks, significant reductions in rotations during the AmIR test (vs. pre-transplantation, *p* < 0.001)	a33	Nishimura et al. (2016)
Rat (6-OHDA)	vmDA progenitor (CORIN^+^ sorted)_Str	AmIR	After 4 months, significant reductions in rotations during the AmIR test (*p* < 0.001)	a34	Kikuchi et al. (2017b)
	vmDA progenitor (PD-hiPSC-derived, CORIN^+^ sorted)_Str	AmIR	After 4 months, significant reductions in rotations during the AmIR test (*p* < 0.01)		
Rat (6-OHDA)	vmDA progenitor (CORIN^+^ sorted)_Str	AmIR	After 16 weeks, significantly decreased rotations in the AmIR test (*p* < 0.001)	a35	Samata et al. (2015)
Monkey (MPTP)	NSC_SN & Str	Healthy behavior score (similar to UPDRS), Parkscore	After 12 months, a significant decrease in the Parkscore was observed in the low-dose (10 M cells injected) group (vs. pre-transplantation, *p* < 0.0143; vs. control, n.s.), whereas there was no significant difference between the healthy behavior scores of the low-dose and control groups	a36	Gonzalez et al. (2016)
Monkey (MPTP)	DA progenitor_Str	NRS, SM, raisin pick-up	After 6 months, a slight improvement in the NRS, accompanied by a 25.0% increase in the large-sized movements, a 25.4% increase in the medium-sized movements, and a 10.8% increase in the total amount of movement, compared to pre-transplantation (no statistical analysis data available); quicker grasping of the raisin with the right arm and quicker retraction of the right arm during the raisin pick-up test (vs. pre-transplantation, n.s.)	a37	Kikuchi et al. (2011)
Monkey (MPTP)	vmDA progenitor_Str	NRS, SM	After 3 months, significant NRS improvement (*p* < 0.001) and increased SM levels (n.s.) were observed exclusively in monkeys with DIV42-cell-transplants	a38	Doi et al. (2012)
Monkey (MPTP)	vmDA progenitor (CORIN^+^ sorted)_Str	NRS, SM	After 12 months, transplanted monkeys showed significantly higher NRS recovery (53.6 ± 8.5% improvement in healthy-cell-transplanted group, *p* < 0.0001; 41.7 ± 14.4% improvement in PD-cell-transplanted group, *p* < 0.01) and increased SM (*p* = 0.0016 [threshold: 5,000 px per 0.033 s] or *p* = 0.0007 [threshold: 10,000 px per 0.033 s] in linear regression analysis of moving time)	a39	Kikuchi et al. (2017a)

AmIR, Amphetamine/methamphetamine-induced rotation. ApIR, Apomorphine-induced rotation; DA, dopamine; DIV, days *in vitro*. n. s., not significant. NRS, neurological rating scale; NSC, neural stem cell. Parkscore, Parkinsonian summary score. SM, spontaneous movement; SN, substantia nigra. Str, striatum.

### 3.5 Transplantation protocols and immune considerations in animal models of PD

In the realm of PD cell therapy, transplantation of vmDA progenitor cells is carried out using a variety of protocols and in different animal models (Table 2). A study has shown that transplanting immature vmDA progenitors results in a higher percentage of TH-positive cells compared to transplanting more mature vmDA progenitors (de Luzy et al., 2021). Various animal models, including rodents and primates, are used for these transplantation experiments. To prepare a rodent model for PD transplantation, 6-OHDA is administered into specific brain regions such as the medial forebrain bundle (MFB), striatum, or substantia nigra (SN). In primate models, MPTP is used for induction. Among rodents, rats are used more frequently than mice, while monkeys are the common choice for primate models. The most common rat model involves transplanting donor cells into the striatum or SN, whereas in the primate model, cells are usually injected into the putamen, a component of the striatum. The number of transplanted cells varies with the size of the animal’s brain; for example, 0.012–0.2 million (M) cells are transplanted into mouse models, 0.001–1.2 M into rat models, and 3.75–20 M into monkey models (Table 2). In rodent models, cells are typically aliquoted and injected either once or twice into the target region (Ben-Hur et al., 2004; Schulz et al., 2004; Brederlau et al., 2006; Sonntag et al., 2007; Cho et al., 2008; Cai et al., 2010; Hargus et al., 2010; Cho et al., 2011; Kriks et al., 2011; Rhee et al., 2011; Ogura et al., 2013; Doi et al., 2014; Grealish et al., 2014; Effenberg et al., 2015; Gonzalez et al., 2015; Grealish et al., 2015; Han et al., 2015; Komatsu et al., 2015; Samata et al., 2015; Steinbeck et al., 2015; Katsukawa et al., 2016; Nishimura et al., 2016; Samata et al., 2016; Adil et al., 2017; Kikuchi et al., 2017b; Wakeman et al., 2017; Xia et al., 2017; Cardoso et al., 2018; Fathi et al., 2018; Adler et al., 2019; Chang et al., 2019; Leitner et al., 2019; Zygogianni et al., 2019; Francis et al., 2020; Gantner et al., 2020; Goggi et al., 2020; Hoban et al., 2020; Miyawaki et al., 2020; Mousavinejad et al., 2020; Schweitzer et al., 2020; Song et al., 2020; Tiklova et al., 2020; Kim et al., 2021b; de Luzy et al., 2021; Piao et al., 2021; Xiong et al., 2021). Conversely, in primate models, all cells may be injected at once into the target region, or they may be aliquoted and administered into various regions of the putamen (Kikuchi et al., 2011; Doi et al., 2012; Wakeman et al., 2014; Gonzalez et al., 2016; Kikuchi et al., 2017a).

Post-transplantation cell loss can occur due to immune responses (Tabar et al., 2008; Morizane et al., 2017). To mitigate this, immunosuppressive drugs like cyclosporin A and FK506 are often administered pre- and post-transplantation (Ben-Hur et al., 2004; Schulz et al., 2004; Brederlau et al., 2006; Sonntag et al., 2007; Geeta et al., 2008; Cai et al., 2010; Hargus et al., 2010; Cho et al., 2011; Kikuchi et al., 2011; Kriks et al., 2011; Rhee et al., 2011; Doi et al., 2012; Grealish et al., 2014; Wakeman et al., 2014; Effenberg et al., 2015; Gonzalez et al., 2015; Han et al., 2015; Komatsu et al., 2015; Samata et al., 2015; Gonzalez et al., 2016; Samata et al., 2016; Adil et al., 2017; Kikuchi et al., 2017a; Kikuchi et al., 2017b; Wakeman et al., 2017; Cardoso et al., 2018; Fathi et al., 2018; Adler et al., 2019; Chang et al., 2019; Hoban et al., 2020). Alternatively, immunodeficient animal models can be used to reduce cell loss due to immune responses (Ogura et al., 2013; Grealish et al., 2015; Steinbeck et al., 2015; Katsukawa et al., 2016; Nishimura et al., 2016; Niclis et al., 2017; Leitner et al., 2019; Zygogianni et al., 2019; Francis et al., 2020; Gantner et al., 2020; Goggi et al., 2020; Miyawaki et al., 2020; Mousavinejad et al., 2020; Schweitzer et al., 2020; Song et al., 2020; Tiklova et al., 2020; Kim et al., 2021b; de Luzy et al., 2021; Piao et al., 2021; Xiong et al., 2021). Regardless of the method used, both approaches have been shown to decrease the number of cells lost to immune reactions post-transplantation. Ultimately, the transplanted donor cells integrate, differentiate, and function as mature vmDA neurons, leading to an improvement in motor symptoms in animal models of PD (Table 3).

### 3.6 Impact of cell sorting on the efficacy and safety of vmDA progenitor cell transplantation

Before transplanting vmDA progenitor cells into animal models of PD, research practices have varied. Some groups sort the vmDA progenitor cells using specific markers, while others do not. Importantly, both groups of researchers used the same markers for *in vitro* identification to confirm the differentiation of the transplanted cells into vmDA neurons *in vivo* (Chang et al., 2019; Gantner et al., 2020; Goggi et al., 2020; Song et al., 2020; Xiong et al., 2021). When vmDA progenitor cells are not sorted prior to transplantation, undesirable lineage differentiation may occur post-transplantation (Cai et al., 2010; Rhee et al., 2011; Wakeman et al., 2017; Kim T. W. et al., 2021). Additionally, unsorted donor cell populations may contain proliferating neural stem cells (NSCs) and hPSCs, raising the risk of teratoma formation (Brederlau et al., 2006; Cai et al., 2010). To minimize these risks, some research groups have used cell sorting with specific markers such as CORIN^+^, LRTM1^+^, CNTN2^+^, LMX1A^+^, and PSA-NCAM^+^ (Table 2). Among these, CORIN, LRTM1, and CNTN2 are specifically used as vmDA progenitor markers, with CORIN being most frequently employed (Doi et al., 2014; Samata et al., 2015; Nishimura et al., 2016; Kikuchi et al., 2017a; Kikuchi et al., 2017b; Miyawaki et al., 2020). Experiments involving CORIN^+^-sorted cells have shown significant improvements in outcomes. Jun Takahashi’s group demonstrated a 96% increase in the number of TH^+^ cells per graft when using CORIN^+^-sorted cells compared to unsorted cells. In addition, CORIN^+^-sorted cells also showed an 18% increase in the proportion of TH^+^ cells among NEUN^+^ cells and led to a smaller average graft size (unsorted, 35.0 ± 37.5 mm^3^; sorted, 3.4 ± 2.9 mm^3^), thereby indicating improved precision and efficacy of the transplantation procedure. Moreover, the number of proliferating cells and the number of serotonin^+^ cells were decreased in CORIN^+^-sorted donor cells (Doi et al., 2014). Post-transplantation functionality of the engrafted cells was assessed using [^18^F]DOPA-PET imaging. Results indicate a substantial increase in dopamine synthesis in the putamen of the striatum over time, affirming the functionality of transplanted cells (Kikuchi et al., 2017a).

When cells were sorted using LRTM1, a distinct vmDA surface marker, there was an increase of 50 ± 3% in FOXA2+ cells, 31 ± 0.8% in NURR1+ cells, and 289% in TH + cells after transplantation. Importantly, the graft size was also reduced in these cases (Samata et al., 2016). When the sorting process was conducted using CNTN2 as the marker, the proportions of both TH+ and DAT + cells saw a rise of approximately 4% following transplantation (Fathi et al., 2018). The maturation of the engrafted cells into vmDA neurons was further confirmed through the observation of mature neuron morphology, including attributes such as neurite extension, arborization, and branching (Nishimura et al., 2016; Kikuchi et al., 2017a; Kikuchi et al., 2017b; Adler et al., 2019; Gantner et al., 2020; Xiong et al., 2021).

### 3.7 Evaluation methods for assessing cell transplantation efficacy in animal models of PD

In evaluating the efficacy of cell transplantation therapies for PD in animal models, a diverse range of behavioral tests have been employed to assess motor function (Table 3). Among the most widely used in rat PD models are the amphetamine/methamphetamine-induced rotation test (Ben-Hur et al., 2004; Brederlau et al., 2006; Sonntag et al., 2007; Cho et al., 2008; Cai et al., 2010; Hargus et al., 2010; Swistowski et al., 2010; Kriks et al., 2011; Rhee et al., 2011; Doi et al., 2014; Grealish et al., 2014; Grealish et al., 2015; Samata et al., 2015; Nishimura et al., 2016; Samata et al., 2016; Kikuchi et al., 2017b; Niclis et al., 2017; Wakeman et al., 2017; Cardoso et al., 2018; Chang et al., 2019; Gantner et al., 2020; Goggi et al., 2020; Song et al., 2020; Tiklova et al., 2020; Kim et al., 2021b; de Luzy et al., 2021; Piao et al., 2021) and the apomorphine-induced rotation test (Ben-Hur et al., 2004; Cho et al., 2008; Geeta et al., 2008; Hargus et al., 2010; Cho et al., 2011; Han et al., 2015; Samata et al., 2016; Wakeman et al., 2017; Fathi et al., 2018), cited extensively across numerous studies. In addition, the cylinder test (Hargus et al., 2010; Cho et al., 2011; Kriks et al., 2011; Samata et al., 2015; Fathi et al., 2018; Gantner et al., 2020; Song et al., 2020; Tiklova et al., 2020; Xiong et al., 2021) and the stepping test (Ben-Hur et al., 2004; Cho et al., 2008; Hargus et al., 2010; Kriks et al., 2011; Song et al., 2020) have served as supplementary methods for understanding the behavioral ramifications of the treatment. Beyond these common approaches, alternative methods have also been explored, including but not limited to forelimb placing (Ben-Hur et al., 2004; Geeta et al., 2008), the corridor test (Song et al., 2020), and the rotarod test (Han et al., 2015; Francis et al., 2020; Xiong et al., 2021). Astonishingly, the majority of these studies (Ben-Hur et al., 2004; Sonntag et al., 2007; Cho et al., 2008; Geeta et al., 2008; Hargus et al., 2010; Swistowski et al., 2010; Cho et al., 2011; Kriks et al., 2011; Rhee et al., 2011; Doi et al., 2014; Grealish et al., 2014; Grealish et al., 2015; Han et al., 2015; Samata et al., 2015; Nishimura et al., 2016; Samata et al., 2016; Kikuchi et al., 2017b; Niclis et al., 2017; Wakeman et al., 2017; Fathi et al., 2018; Chang et al., 2019; Gantner et al., 2020; Song et al., 2020; Tiklova et al., 2020; Kim et al., 2021b; de Luzy et al., 2021; Piao et al., 2021), with the singular exception of one paper (Brederlau et al., 2006), reported significant improvements in motor symptoms following cell transplantation (Table 3). The mouse PD models have similarly been the subject of various testing paradigms, such as the amphetamine/methamphetamine-induced rotation test (Kriks et al., 2011; Steinbeck et al., 2015; Leitner et al., 2019; Xiong et al., 2021), apomorphine-induced rotation test (Francis et al., 2020), corridor test, (Steinbeck et al., 2015), cylinder test (Francis et al., 2020; Xiong et al., 2021), and rotarod test (Francis et al., 2020; Xiong et al., 2021). In these cases, the results also overwhelmingly favored an improvement in motor symptoms post-transplantation (Table 3). Moving to more complex animal models, nine studies have been conducted utilizing monkey models (Tables 2, 3). A subset of four out of these nine studies specifically evaluated the behavior of the Monkey PD model, and they are summarized in Table 3. Notably, in studies led by Jun Takahashi’s group, a comprehensive neurologic rating scale along with video-based analysis of spontaneous movements were the primary evaluation tools. In every case within this framework, symptoms showed improvement post-cell transplantation (Kikuchi et al., 2011; Doi et al., 2012; Kikuchi et al., 2017a). Another research group has used alternative evaluation metrics such as the Parkscore and Healthy Behavior Score, and this study has likewise confirmed symptom improvement (Gonzalez et al., 2016). Significantly, upon a thorough review of existing research, a discernible trend begins to emerge: there appears to be a plausible correlation between cell sorting prior to transplantation and enhanced behavioral outcomes compared to unsorted cells. This pattern has been observed across a range of animal models, adding credence to the idea that quality control measures like cell sorting may hold potential value in augmenting the efficacy of cell transplantation techniques for PD.

## 4 Progress and challenges in the clinical application of stem cell therapies for PD

Advancements in the field of stem cell therapy for PD have come a long way, from initial *in vitro* generation of vmDA progenitor cells to successful transplantation in animal models, and finally to clinical trials in human patients. A 2019 case-series study spearheaded by Madrazo et al. demonstrated the potential for using human fetal NPCs as a treatment for PD (Madrazo et al., 2019). The study involved injecting NPCs into the dorsal putamina of patients while administering cyclosporine A to mitigate the risk of immune rejection. Longitudinal evaluations over a 4-year period, encompassing neurological, neuropsychological, and brain imaging analyses, revealed motor improvements in all but one of the seven patients followed, with PET scans indicating a trend of increased dopamine activity in the midbrain.

Another milestone was achieved by Jun Takahashi’s research group, pioneers in hiPSC-based therapy for PD (Takahashi, 2020). Beginning their clinical trials in August 2018, they have effectively differentiated DA neurons from hiPSCs. To improve transplantation success rates, they developed a method for selecting DA progenitor cells using the specific vmDA floor plate marker, CORIN (Table 1, Table 2). Confirmations were secured that these CORIN^+^-sorted cells not only survived both *in vitro* and *in vivo* conditions but also functionally differentiated into vmDA neurons, as evidenced in various animal models of PD (Doi et al., 2014; Kikuchi et al., 2017a; Doi et al., 2020). The team also validated the safety and effectiveness of their specialized hiPSC line, QHJI-01, focusing on FOXA2^+^TUJ1^+^ cells as the final product (Doi et al., 2020). The threshold for the final cell population designated for transplantation was established at 80%, with the remainder of the population comprised of midbrain glial cells, which play a supportive role for DA neurons. To minimize potential adverse effects, rigorous QC ensured the absence of hiPSCs expressing OCT3/4 and TRA-2-49/6E markers, as well as NPCs expressing SOX1 and PAX6. The study, designed as a single-arm, non-randomized, open-label Phase I/II trial, initiated patient recruitment in August 2018. The first patient intake session took place at Kyoto University Hospital in October of the same year. To mitigate the risk of immune rejection post-transplantation, patients were administered tacrolimus for a period of 1 year.

When utilizing cells obtained from a different individual for transplantation, there arises the issue of immunocompatibility (Kordower et al., 2008). Immunocompatibility issues stemming from HLA mismatches can lead to the transplanted cells being targeted by the recipient’s immune system (Kordower et al., 1997). Consequently, transplant recipients must employ immunosuppressive agents to suppress immune responses (Morizane and Takahashi, 2021). Prolonged use of immunosuppressive agents can give rise to infections and other immunological complications (Lopez et al., 2006).

Another notable contribution came from a study by Schweitzer et al., in which autologous transplantation of hiPSC-derived DA neurons was performed without immunosuppression (Schweitzer et al., 2020). These cells survived for up to 2 years post-transplantation, and clinical measures showed that PD symptoms had either stabilized or improved during an 18–24-month follow-up period. Internationally, both China and Australia are also conducting PD cell transplantation trials using hESCs, as ClinicalTrials.gov numbers NCT03119636 and NCT02452723 indicate. In addition to the aforementioned studies, various clinical trials utilizing hPSC-derived dopaminergic cells are currently underway (Barker et al., 2017).

Despite these advances, challenges still loom large, such as safety concerns associated with viral vectors used in reprogramming hiPSCs (Ma et al., 2014; Kang et al., 2016). However, recent developments like the protocol by Guan et al., which replaces viruses with chemicals for generating clinical-grade hiPSCs, mark a promising step towards overcoming these obstacles (Guan et al., 2022). In summary, although hPSC-based cell therapies are advancing toward clinical applications, several challenges persist, such as safety concerns and potential complications arising from the use of adult or embryonic cells, as well as immunocompatibility issues. Nevertheless, the momentum gained from these groundbreaking endeavors holds promise for a more effective and hopeful future in the treatment of PD.

## 5 Discussion

The potential of human pluripotent stem cells (hPSCs) to differentiate into ventral midbrain dopaminergic (vmDA) progenitors presents an exciting avenue for the treatment of Parkinson’s Disease (PD). Various protocols have been developed to guide this differentiation, often achieving high yields of vmDA progenitors and neurons. However, the challenge lies in the concomitant generation of cells from other lineages (Table 1), which risks the introduction of undesired cell types during transplantation. Current QC methods for ensuring the lineage-specificity of the generated cells are not yet fully reliable. Jun Takahashi’s group sought to overcome this hurdle by using the surface marker CORIN to sort vmDA progenitors (Table 2, Table 3). Yet, subsequent research indicated that CORIN expression is not confined to the caudal vmDA progenitor region, raising concerns about the specificity of this sorting mechanism (Kirkeby et al., 2017). The clinical implications of these challenges are significant. Transplanting cells with unconfirmed lineage can lead to complications such as tumorigenicity and dyskinesias, emphasizing the need for rigorous QC procedures. While current differentiation protocols show promise, the lack of precise lineage-verification methods raises questions about their readiness for clinical applications. Emerging technologies like nanoprobe-based lineage verification may offer a solution to these challenges. The development of more specific surface markers or advanced detection techniques could enable the selective harvesting of vmDA progenitors, reducing the risk of adverse effects and potentially enhancing therapeutic efficacy.

In conclusion, while hPSC-derived vmDA progenitors present an encouraging path for PD treatment, a robust framework for lineage verification is imperative for ensuring the safety and effectiveness of this promising therapeutic approach. Bridging the gap between preclinical research and clinical practice remains a crucial task for the field, demanding continued interdisciplinary efforts to overcome these limitations.
